# Resveratrol: A Multifaceted Guardian against Anxiety and Stress Disorders—An Overview of Experimental Evidence

**DOI:** 10.3390/nu16172856

**Published:** 2024-08-26

**Authors:** Vadim E. Tseilikman, Olga B. Tseilikman, Oleg N. Yegorov, Alina A. Brichagina, Marina N. Karpenko, David V. Tseilikman, Vladislav A. Shatilov, Maxim S. Zhukov, Jurica Novak

**Affiliations:** 1 Scientific and Educational Center ‘Biomedical Technologies’, School of Medical Biology, South Ural State University, 454080 Chelyabinsk, Russia; 2 Zelman Institute of Medicine and Psychology, Novosibirsk State University, 630090 Novosibirsk, Russia; 3 Faculty of Fundamental Medicine, Chelyabinsk State University, 454001 Chelyabinsk, Russia; 4 Pavlov Department of Physiology, Institute of Experimental Medicine, 197376 Saint Petersburg, Russia; 5 Center for Artificial Intelligence and Cybersecurity, University of Rijeka, 51000 Rijeka, Croatia

**Keywords:** resveratrol, anxiety, PTSD, glucorticoids, monoamines, neuroinflammation, limbic–hypothalamus–pituitary axis

## Abstract

The medicinal properties of resveratrol have garnered increasing attention from researchers. Extensive data have been accumulated on its use in treating cardiovascular diseases, immune system disorders, cancer, neurological diseases, and behavioral disorders. The protective mechanisms of resveratrol, particularly in anxiety-related stress disorders, have been well documented. However, less attention has been given to the side effects of resveratrol. This review explores not only the mechanisms underlying the anxiolytic effects of resveratrol but also the mechanisms that may lead to increased anxiety following resveratrol treatment. Understanding these mechanisms is crucial for enhancing the efficacy of resveratrol in managing anxiety disorders associated with stress and PTSD.

## 1. Introduction

**Figure 1:**
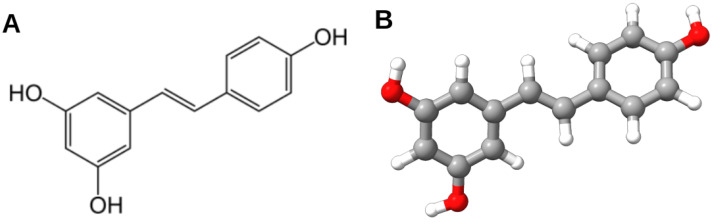
Two-dimensional (**A**) and 3D (**B**) representations of resveratrol’s molecular geometry.

Resveratrol (RES) (Figure 1) is a polyphenol classified as a stilbene, resembling diethylstilbestrol, a synthetic estrogen [1]. Resveratrol exists in both *trans*- and *cis*-isomeric forms and is abundant in red wine and grape skins, seeds, and dried plant roots [2]. Over recent decades, numerous beneficial effects of RES have been reported, including antidiabetic, antiatherogenic, antihypertensive, and anticancer properties. Many of these effects are particularly relevant to stress-related diseases [3].

RES’s low oral bioavailability and short biological half-life limit its therapeutic benefits [4]. Chow et al. [5] conducted a significant study involving 11 men and 31 women to examine the impact of RES on phase I isoenzymes (cytochrome P450, CYPs) and phase II detoxification enzymes after a daily intake of 1 g RES for one month. The phenotypic index for CYP3A2, CYP2D6, and CYP2C9 decreased by 50%, 75%, and 175%, respectively, while it increased by 20% for CYP1A2. Conversely, RES’s inhibition of CYP isoenzymes could affect the bioavailability and metabolism of various drugs, including cancer treatments. Further research is necessary to determine the long-term effects of high-dose RES on the CYP system, particularly when combined with therapeutic drugs. Without addressing safety concerns, RES’s future as a multitarget pharmacological agent remains uncertain.

RES’s multitarget pharmacological effects make it an attractive candidate for addressing behavioral disorders, particularly anxiety disorders, due to the involvement of numerous molecular targets in their pathogenesis.

Stress and anxiety play crucial roles in the adaptation process. Stress is an adaptive response aiding in maintaining homeostasis in challenging situations, while anxiety acts as a preventive measure against exposure to dangerous situations. However, prolonged stress can lead to stress-related disorders, including anxiety disorders. Anxiety, as an emotional state, serves as the first line of defense in anticipation of danger signals from the environment [6]. Anxiety may be accompanied by fear, defined as the emotional response to imminent threat. Anxiety-like behavior can trigger stress as a physiological reaction to a stimulus, while anxiety-related disorders are behavioral complications of stress, with symptoms such as those seen in post-traumatic stress disorder (PTSD) [7].

Recent studies have identified multiple brain areas involved in stress and anxiety responses, including the hypothalamus, amygdala, prefrontal cortex, ventral tegmental area (VTA)–nucleus accumbens (NAc) pathways of the mesolimbic system, locus coeruleus, and raphe nucleus. Persistent anxiety-like behavior is associated with neuroinflammation, oxidative stress, and reduced neuroplasticity, which are closely interconnected [8,9,10,11,12,13,14].

RES exhibits neuroprotective properties, primarily regulating stress-related anxiety behavior. Its neuroprotective potential lies in its ability to target multiple signaling pathways involving AMP-activated protein kinase (AMPK), neurotrophins such as brain-derived neurotrophic factor (BDNF), transcription factors such as cAMP response element-binding protein (CREB), nuclear factor kappa-light-chain-enhancer of activated B cells (NF-κB), nuclear factor erythroid 2-related factor 2 (Nrf-2), and others. Notably, many of these targets are activated through sirtuin 1 (SIRT1) [15]. Sirtuins, nicotinamide adenine dinucleotide (NAD)-dependent deacetylases, are involved in stress responses and are considered longevity genes, with SIRT1 being the main intracellular target of RES [16]. SIRT1 regulates histone deacetylase (HDAC) activity, modulating the expression of multiple genes [17]. The neuroprotective potential of RES may improve anxiety behavior by correcting the pathogenesis of stress-related anxiety disorders. RES has been shown to ameliorate anxious behavior by correcting oxidative stress, mitochondrial dysfunction, and neuroinflammation; improving neuroplasticity; and restoring neurotransmitter balance in various brain areas [18].

In this review, we summarize experimental data on the effectiveness of RES in treating stress-related anxiety-like behavior and discuss the common mechanisms underlying its protective effects at the cellular and system levels.

## 2. Exploring the Neuroprotective Potential of Resveratrol

RES has demonstrated significant neuroprotective potential through its antioxidant properties, suppression of cell death, enhancement of mitochondrial function, and promotion of neuroplasticity (Table 1). These effects contribute to reducing neuroinflammation and improving behavioral disorders. Studies utilizing in silico analyses, neuronal cultures, and animal models have identified key molecular targets, including genes such as prostaglandin-endoperoxide synthase 2 (PTGS2), glutathione reductase (GSR), and BDNF, as well as transcription factors like Nrf-2 and peroxisome proliferator-activated receptor γ (PPARγ). RES regulates these targets by upregulating beneficial genes and downregulating pro-inflammatory genes while also influencing specific microRNAs, thereby supporting its neuroprotective effects.

**Figure 2:**
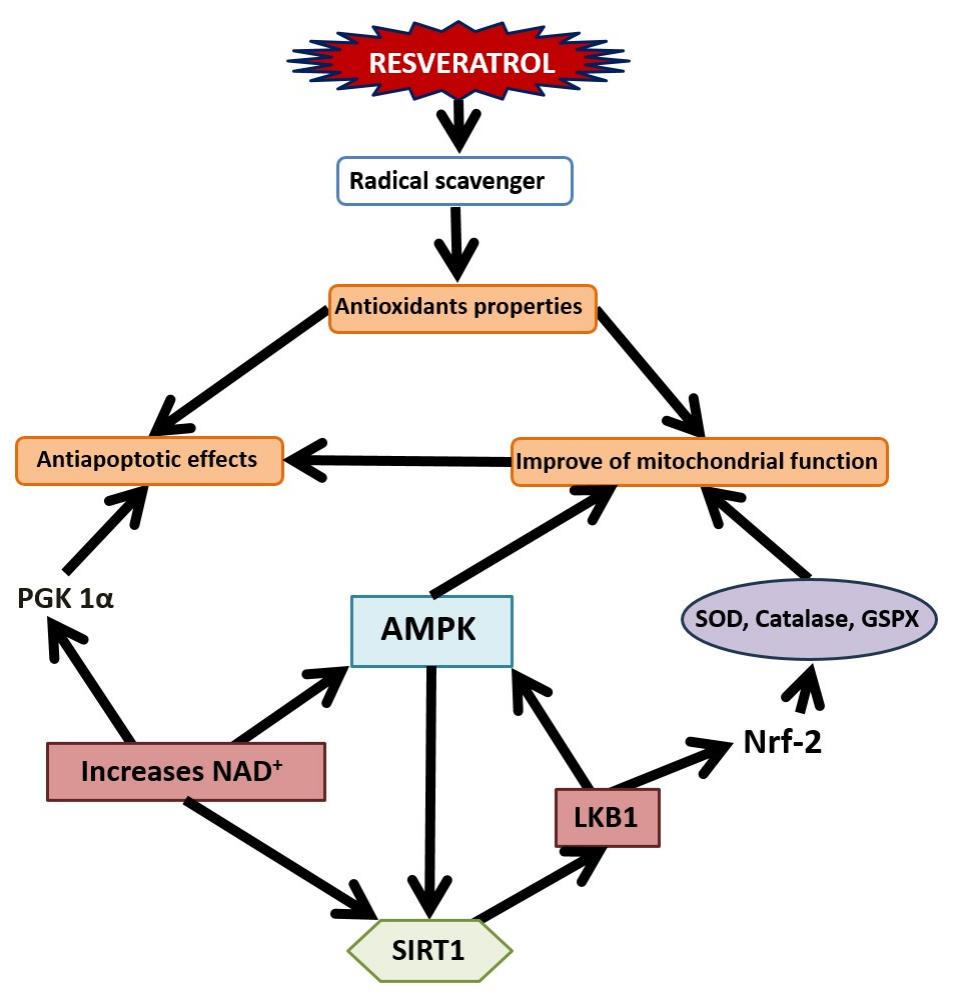
Molecular pathways involved in the neuroprotective effects of resveratrol.

Despite the presence of multiple molecular targets for RES, sirtuin (SIRT) proteins stand out due to the crucial role of SIRT1 in maintaining genome integrity, as evidenced by studies on SIRT1 knockout mice [28]. Several cellular signaling pathways are modulated by RES in a SIRT-dependent manner (see Table 1). SIRT1 exerts broad effects by regulating cellular senescence, proliferation, metabolism, DNA repair, apoptosis, and cell survival. In silico data suggest that RES can directly bind to SIRT proteins with deacetylase activity towards nucleosomes [29,30,31]. Recent studies have elucidated the mechanism of RES action in activating SIRT1 enzyme (Figure 2). However, in addition to SIRT1, some effects of RES treatment are mediated by sirtuin 3 (SIRT3), a major mitochondrial sirtuin that plays a crucial role in regulating mitochondrial viability [32,33].

In silico studies suggest that RES targets the *N*-terminal domain (NTD) of SIRT1 by interacting with three active sites, a finding supported by in vitro experiments [34]. RES also enhances SIRT1 activity by promoting its binding with lamin A, aiding in nuclear matrix localization [35]. The activation of SIRT1 by RES is closely linked to the adenosine monophosphate kinase (AMPK) pathway, where AMPK increases nicotinamide phosphoribosyltransferase (NAMPT) expression, raising intracellular NAD levels and further activating SIRT1 [36]. SIRT1, in turn, activates AMPK by deacetylating liver kinase B1 (LKB1), which phosphorylates AMPK on threonine 172 in the α subunit. The LKB1-STRAD (STE20-related adapter)–MO25 (mouse protein 25) complex is crucial for LKB1’s kinase activity in metabolic signaling pathways, with RES upregulating LKB1 activity [37]. Additionally, activated SIRT1 deacetylates peroxisome proliferator-activated receptor-gamma coactivator-1-alpha (PGC1α), a key regulator of reactive oxygen species (ROS) production and detoxification. While PGC1α is linked to mitochondrial biogenesis and increased ROS production, it also enhances the expression of detoxifying enzymes, thus controlling the oxidative defense system [36,38,39].

Metabolomics analysis revealed alterations in glucose metabolism upon deletion of neuronal SIRT1, accompanied by transcriptional changes in glucose metabolism machinery [40,41]. In SIRT1 knockout mice, RES increased the glycolytic rate in an AMPK-dependent manner via the protein kinase A (PKA)/LKB1/AMPK pathway [42].

Overall, SIRT1 and AMPK occupy upstream positions in signaling cascades that mediate RES neuroprotective potential, while enzymes such as extracellular signal-regulated kinase 1/2 (ERK1/2), monoamine oxidase (MAO), and transcription factors and neurotrophins such as BDNF, CREB, Nrf-2, mammalian target of rapamycin (mTOR), Forkhead box O (FOXO), NF-κB, and others are downstream targets [43].

In various types of neuronal cultures, it has been demonstrated that RES’s protective effects against mitochondrial dysfunction, oxidative stress, and apoptosis are promoted by enhanced SIRT1 expression [44]. Therefore, SIRT drives different molecular pathways and mediates various protective effects of RES treatment. Numerous data suggest that the antiapoptotic properties of RES treatment are closely linked to antioxidant enzymes. The antioxidant properties of RES in neuronal and glial cells are performed via the SIRT1/Nrf-2 pathway [45], where Nrf-2 acts as a transcription factor triggering the expression of numerous antioxidant enzymes. RES has been shown to reduce oxidative stress in primary neuronal cultures by increasing the activities of antioxidant enzymes, such as superoxide dismutase (SOD), catalase, glutathione reductase, and non-enzymatic antioxidants such as tocopherol, ascorbic acid, and glutathione, while simultaneously attenuating the accumulation of lipid peroxide levels [22]. RES treatment enhances Nrf-2 and its downstream targets. RES has also been indicated as a scavenger of free radicals [46]. RES’s antioxidant properties also occur via interaction with complex III of the respiratory chain, assuming a role as a radical scavenger and therefore a suppressor of radical formation in the mitochondria. In models of oxygen and glucose deprivation/reoxygenation, RES treatment at different times increased neuronal viability and inhibited neuronal apoptosis in vitro, at least in part, by enhancing the activation of the Nrf-2 signaling pathway [23]. It is suggested that in neuronal cells, RES’s antiapoptotic effects are at least partially related to the antioxidant properties of this polyphenol [47]. RES-loaded nanoparticles (RES-NPs) based on poly(*N*-vinylpyrrolidone)-b-poly(ε-caprolactone) polymer have a protective effect against hydrogen peroxide-induced oxidative stress and apoptosis in rat cortical cell culture [48].

Zebrafish, used as a model organism for drug screening, have been utilized to assess whether RES’s neuroprotective effects observed in vitro can be reproduced in vivo. Importantly, RES offers protection against *N*-methyl-*D*-aspartate receptor (NMDA)-induced retinal damage [49]. RES glucosyl- and glucosyl-acyl-derivatives showed lower neurotoxicity in zebrafish embryos [50]. In a 3-nitropropionic acid animal model of Huntington’s disease (HD), RES derivatives delayed the onset and reduced the severity of HD-like symptoms, improved locomotor activity, and protected against weight loss, with simultaneous enhancement of superoxide dismutase 2 (SOD2) expression in brain tissue and a decrease in circulating levels of interleukin-6 (IL-6), respectively [51]. RES treatment alleviates neurological deficits, reduces cell death, and increases hematoma clearance in the intracerebral hemorrhage experimental model in SIRT3 knockout mice. RES also effectively suppresses CD16^+^ microglia/macrophage activation and decreases tumor necrosis factor (TNF) release [52]. The antiapoptotic properties of RES treatment are mediated via poly [ADP-ribose] polymerase 1 (PARP-1), downstream modulating DNA fragmentation and the expression of several genes implicated in the apoptotic cascade, such as Bax, B-cell lymphoma-2 (Bcl-2), and cleaved caspase 3 (CASP3). RES also prevents the proapoptotic increase in p53 [19,20]. RES exerted antiapoptotic effects in models of 1-methyl-4-phenyl pyridinium (MPP(+))-induced cytotoxicity of dopaminergic neurons [36] and in the model of amphetamine-mediated neurotoxicity of dopaminergic neurons isolated from the midbrain of mouse embryos [24]. Moreover, in the aforementioned studies, RES exclusively acted as an antioxidant [26]. In the model of glutamate excitotoxicity, therapeutic efficacy of RES co-treatment against glutamate-induced radical formation in RES-treated cultures was shown [25,53]. Simultaneously, in these studies, an elevation in the number of dopaminergic neurons in the RGC5 neuronal cells was revealed [54]. RES reduced apoptosis, decreased oxidative status, and alleviated mitochondrial damage in amyloid β-peptide (1-42) (Aβ1-42)-treated PC12 cells [55]. Meanwhile, the antiapoptotic effects of resveratrol on damage to mouse cortical neurons induced by β-amyloid through the activation of the protein kinase B (PKB, also known as Akt1) pathway were noted [56]. RES exerted neuroprotective effects against mitochondrial dysfunction due to downregulation of caspase 3 and 9 activity in SH-SY5Y cells [27].

Neuroprotective effects of RES are strongly associated with improved mitochondrial function. RES enhances mitochondrial function via the activation of the SIRT1/AMPK/ PGC-1α pathway [27]. Specifically, RES targets SIRT1, which in turn upregulates AMPK through LKB1. AMPK promotes the protective effects of PGC-1α on mitochondria, mediating the antiapoptotic effects of RES. Independent of PGC-1α, RES’s antiapoptotic effects may also be facilitated via Nrf-2, a key antioxidant transcriptional factor, resulting in increased transcription of antioxidant enzymes such as superoxide dismutase, catalase, and glutathione peroxidase (GSPX) [57,58]. Additionally, RES acts as a free radical scavenger, as it easily enters mitochondria, further contributing to its antioxidant effects (Figure 2) [59].

Mitochondrial oxidative stress often induces mitochondrial dysfunction. RES has been shown to restore elevated levels of mitochondrial respiration, mtDNA content, and complex IV activity [59,60]. Mitochondrial damage is a well-known cause of apoptosis, and RES, as a SIRT1 activator, promotes mitochondrial function in neuronal cells, [61] accompanied by the increased expression of succinate dehydrogenase (SDH), a mitochondrial enzyme. Notably, this effect is blocked by SIRT1 siRNA [54]. Moreover, RES improves the balance between mitophagy and mitochondrial biogenesis in Corti 1 hair cells via miR-34a/SIRT1 signaling [62]. In the zebrafish retina, RES increases mitochondrial quality and function while simultaneously suppressing Akt/mTOR activity. RES treatment promotes SIRT1, mitochondrial sirtuins, and protein expression and improves mitochondrial DNA repair in the adult zebrafish retina [63].

The improvements in mitochondrial function and the antiapoptotic effects of RES are mediated via the SIRT1/SIRT3/AMPK/PGC-1α pathways. AMPK enhances the expression of nicotinamide phosphoribosyltransferase, which increases intracellular NAD^+^ concentration and activates SIRT1. In turn, SIRT1 promotes AMPK activation by deacetylating and activating LKB1, the upstream activator of AMPK [64]. RES treatment also regulates the interplay between SIRT1 and other signaling pathways, notably upregulating SIRT1 protein by activating LKB1.

Resveratrol exhibits significant neuroprotective effects by influencing brain metabolism, enhancing mitochondrial function, and regulating autophagy, largely through the activation of AMPK.

**Figure 3:**
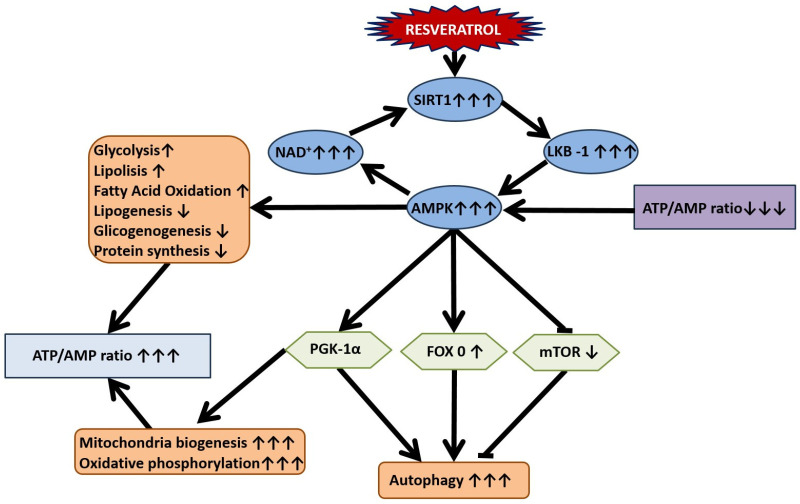
Resveratrol impact on metabolism via SIRT1/AMPK/PGK1-α pathway, indicated by upregulation (upward arrows) or downregulation (downward arrows).

AMPK is a unique enzyme that responds to changes in AMP/ATP levels, playing a crucial role in cellular energy homeostasis [65]. An increase in AMP/ATP levels leads to decreased anabolic (energy-consuming) processes and increased catabolic (energy-producing) processes due to AMPK activation. The metabolic effects of AMPK involve the activation of FOXO-dependent pathways and the inhibition of mTOR-dependent pathways, which together provoke autophagy [66]. This balance between FOXO and mTOR pathways is critical for cellular energy regulation and survival. The SIRT1/AMPK/PGC-1α pathway promotes mitochondrial biogenesis and optimizes AMP/ATP levels, ensuring efficient energy production and utilization in cells (Figure 3) [67].

The AMPK-SIRT1 pathway regulates autophagy by inducing the assembly of autophagosomes through the increased expression of microtubule-associated protein 1A/1B-light chain 3, a protein associated with autophagosome membranes [68]. RES activates AMPK/SIRT1, leading to the induction of autophagy, which enhances the clearance of damaged mitochondria (mitophagy). This process reduces rotenone-induced apoptosis by decreasing the levels of cytochrome C released from injured mitochondria. RES promotes autophagy through the phosphorylation of AMPK [69]. In primary neuronal cultures, RES enhances autophagy, which can be diminished by inhibiting AMPK activation with Compound C, indicating that RES’s protective effect partially depends on the AMPK/autophagy pathway [70]. RES may also activate other mitochondrial-related autophagic pathways, such as the MEK/ERK signaling pathway and the Jun *N*-terminal kinases-associated B-cell lymphoma-2 (JNK/Bcl-2) pathway, contributing to its neuroprotective effects by increasing autophagy and reducing oxidative stress and apoptosis [71]. RES has been shown to mitigate methamphetamine-induced cell apoptosis in models of glutamate excitotoxicity [72].

Neuronal cell cultures are essential for understanding the basic mechanisms underlying the neuroprotective effects of resveratrol. However, since the functioning of neurons is supported by glial cells, it is crucial to consider the effects of RES on glial cells to gain a complete picture of its neuroprotective properties, particularly concerning neuroinflammation and neuroplasticity.

The protective effect of RES against neuroinflammation is primarily based on the regulation of glial cell activity through the downregulation of NF-κB pathways [73]. NF-κB-mediated oxidative stress leads to mitochondrial dysfunction and apoptosis in glial cells. Activated microglia release significant amounts of pro-inflammatory cytokines and neurotoxic mediators, which can contribute to brain injuries [74]. The NF-κB signaling pathway in microglia is implicated in amyloid-β (Aβ) peptide-induced neurodegeneration [75]. The constitutive inhibition of NF-κB signaling in microglia through the expression of a nondegradable IκBα super-repressor blocks neurotoxicity, indicating a pivotal role for microglial NF-κB signaling in mediating Aβ toxicity [75]. Stimulation of microglia with Aβ increases acetylation of RelA/p65 at lysine 310, regulating the NF-κB pathway. Overexpression of the SIRT1 deacetylase and the addition of the SIRT1 agonist resveratrol markedly reduce NF-κB signaling stimulated by Aβ and exert strong neuroprotective effects [75].

RES significantly inhibits hypoxia-induced microglial activation and reduces the subsequent release of pro-inflammatory factors [76]. It also inhibits the hypoxia-induced degradation of IκB-α and phosphorylation of p65 NF-κB protein [76]. Furthermore, RES pretreatment inhibits apoptosis in microglial cells, reduces oxidative stress [77], and inhibits lipopolysaccharide (LPS)-mediated proinflammatory cytokine release in microglia, reducing the p53-caspase-3-dependent mechanism of apoptosis [78]. RES also inhibits LPS- and ATP-activated NLR family pyrin domain containing 3 (NLRP3) inflammasome and protects microglial cells from oxidative stress, proinflammatory cytokine production, and pyroptotic cell death resulting from inflammasome activation [79].

RES inhibits NF-κB signaling and activates the AMPK/SIRT1 pathways. It has been shown to downregulate inflammasome-induced miR-155 expression, and the inhibition of AMPK and SIRT1 pathways significantly reverses RES’s protective effect on miR-155 expression in microglia [79]. RES pretreatment ameliorates cognitive impairment in developing mice exposed to sevoflurane by modulating the SIRT1/NF-κB pathway in microglia [80]. Notably, RES pretreatment reverses sevoflurane-induced SIRT1 inhibition and microglial activation [80]. Sevoflurane is a commonly used inhaled anesthetic. RES also reverses the sevoflurane-induced imbalance of the M1/M2 microglia ratio, as revealed by increased mRNA levels of clusters of differentiation 206 (CD206) and decreased mRNA levels of clusters of differentiation 86 (CD86) and suppressor of cytokine signaling 3 (SOCS3) [80].

SOCS3 is potentially another target for RES because its expression in the brain rapidly increases in response to several pro-inflammatory cytokines [81]. Upon stimulation by cytokines, SOCS3 expression increases, disturbing insulin signaling by inhibiting the tyrosine phosphorylation process of insulin receptor substrat 1 (IRS1). This increases serine phosphorylation of IRS1, resulting in inflammatory effects and neuronal lesions, limiting glucose intake in the brain [82]. SOCS3 is implicated in Aβ peptide-induced neurotoxicity. RES may target SOCS3 via SIRT1/AMPK pathways, supported by data illustrating that AMPK-autophagy activation suppresses neuroinflammation and improves morphine tolerance via the upregulation of SOCS3 by inhibiting miRNA-30a-5p [83].

Autophagy is an adaptive response that preserves cellular viability by removing damaged ultrastructures. RES modulates autophagy via AMPK-dependent signaling, thereby preserving the viability of both neurons and glial cells [84].

**Figure 4:**
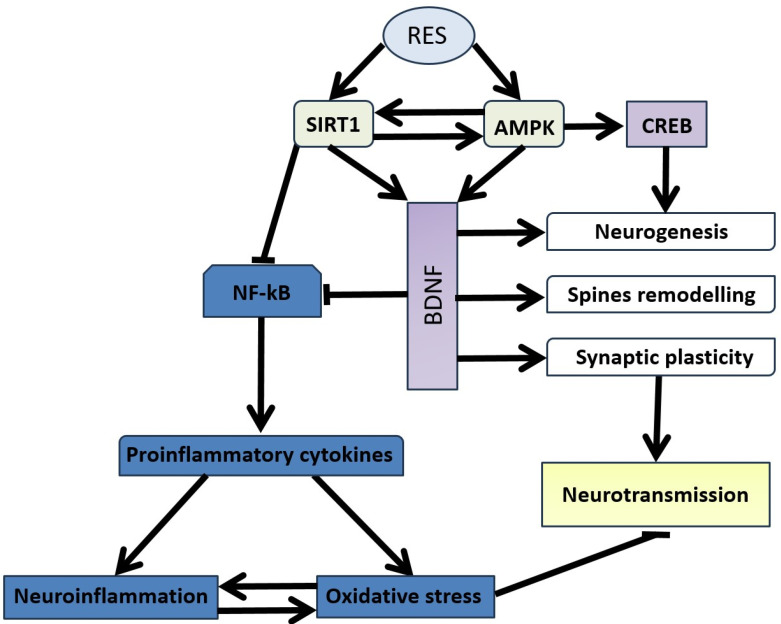
Resveratrol upregulates neuroplasticity and downregulates neuroinflammation.

RES promotes neuroplasticity through the SIRT1/AMPK/CREB/BDNF pathway, enhancing synaptic plasticity and overall neurotransmission (Figure 4) [85]. By downregulating NF-κB, RES limits the release of pro-inflammatory cytokines from glial cells, reducing neuroinflammation and oxidative stress, which in turn improves neurotransmission. This is particularly important in stress-related anxiety disorders, where the restoration of neurotransmission is a crucial protective effect of RES.

BDNF plays a vital role in maintaining neuronal plasticity and regulating mental states. The effects of BDNF are mediated by its receptor, tropomyosin receptor kinase B (TrkB), which influences various types of neurons and glial cells. Numerous studies have shown a mutual interaction between BDNF/TrkB signaling, SIRT proteins, AMPK, neuroinflammation, and oxidative stress. A deficiency in BDNF can exacerbate oxidative stress in neurons by reducing CREB activity and increasing NF-κB binding activity [86,87]. BDNF not only inhibits neuroinflammation but also promotes the release of various neurotrophic factors from astroglia, which are critical for neuroprotection [88].

RES significantly induces the phosphorylation of ERK1/2 and CREB in astroglia, which leads to the increased release of astroglia-derived neurotrophic factors [89]. The interplay between the SIRT1/miR-134 signaling pathway, CREB, and BDNF expression enhances RES’s neuroprotective effect in primary cultured hippocampal neurons [90]. This occurs through the activation of the SIRT1/miR-134 pathway by RES, followed by the upregulation of CREB/BDNF expression in the hippocampus [90].

BDNF, through the BDNF/CREB/AMPK pathway, is a key signaling molecule that links synaptic plasticity and energy metabolism [91]. Additionally, other neurotrophic factors, such as glial cell line-derived neurotrophic factor (GDNF), contribute to the development, maintenance, and survival of neurons, glia, and oligodendrocytes. Since astroglia are a major source of these neurotrophic factors, enhancing astroglia-mediated neurotrophic factor release holds promising potential for treating neurological diseases. Astroglia-derived neurotrophic factors have permissive effects on RES treatment towards lesions in dopaminergic neurons [91]. Recent studies have shown that RES treatment improves synaptic plasticity and alleviates neurotransmitter signaling through the SIRT1/PGC-1α pathway [92].

Dendritic plasticity is a fundamental mechanism of the central nervous system, crucial for synaptic potentiation, memory formation, learning, cognitive abilities, and overall brain function [93]. BDNF plays a significant role in preventing dendritic atrophy by increasing spine density in primary hippocampal neuron cultures [93]. Therefore, the interaction between RES and BDNF may improve dendrite phenotypes in vitro.

In unstimulated cultures, both AMPA receptor subunit (GluR1) and NMDA receptor subunit (NR1) are concentrated in SV2-positive synaptic clusters associated with dendritic shafts and spines [94]. Glutamate-induced neurotoxicity decreases the density of dendritic spines in hippocampal neurons [95]. However, BDNF can prevent glutamate-induced excitotoxicity through pathways such as phosphatidylinositol 3-kinases (PI3K), phospholipase C-γ (PLC-γ), and ERK. Conversely, excessive glutamate can inhibit BDNF expression by activating extrasynaptic NMDA and eukaryotic elongation factor 2 (eEF2) [74].

RES increases dendritic spine density and the expression of postsynaptic density protein 95 (PSD95) and BDNF, ameliorating paclitaxel-induced synaptic damage [96]. Besides BDNF and GDNF, RES also activates ERK1/2 and CREB [89]. The neuroprotective action of RES in vitro is associated with increased levels of SIRT1, CREB phosphorylation (p-CREB), CREB, and BDNF, along with decreased levels of miR-134 [89]. Additionally, RES enhances the expression of synaptic plasticity-associated proteins such as synaptic ras GTPase activation protein (SynGAP), postsynaptic density protein 95 (PSD95), synapsin-1, and synaptogmin-1 in the hippocampus, a process dependent on SIRT1 [96,97].

RES’s ability to ameliorate synaptic plasticity is particularly beneficial in correcting stress-related anxiety disorders, as synaptic stability significantly affects neurotransmitter efficacy. Overall, the neuroprotective properties of RES contribute to its effectiveness in treating anxiety disorders by enhancing synaptic plasticity and improving neurotransmitter function.

The information presented here leads to several key conclusions regarding the neuroprotective effects of RES. At the cellular level, these effects are primarily driven by RES’s ability to enhance neuroplasticity and exert antiapoptotic properties, with the latter being largely attributed to its antioxidant activity. The antioxidant effects of RES not only support mitochondrial function but also improve mitochondrial performance, thereby reducing the risk of neuronal apoptosis. Additionally, RES’s enhancement of neuroplasticity results in dendritic remodeling and improved synaptic function. Furthermore, the neuroprotective effects of RES are associated with global transcriptome remodeling, involving the activation and suppression of specific signaling pathways, as well as its role as a free radical scavenger.

## 3. Stress-Related Anxiety Disorders: State of the Art

Anxiety can be viewed in two ways: as a personality trait and as the main behavioral response to stress [98]. The primary function of anxiety is to avoid dangerous situations during stressful events, with hypervigilance in anticipation of a threat indicating the severity of stress. Recent studies on stress-related anxiety disorders aim to translate their findings into clinical practice. Some research has explored the link between anxiety and nutritional status. Transcriptomic analysis of brain tissues involved in anxiety (hypothalamus, amygdala, and pituitary) has revealed that long-term calorie restriction (CR) alters anxiety-promoting pathways [99]. It was reported that animals experiencing variable periods of food availability developed a selective response to these fluctuations. Two scenarios were reviewed: one short term (typical CR) and the other long term (acting on intergenerational timescales), demonstrating the benefit of having mood and exploratory drive regulated by food availability [99]. CR mice were found to be more anxious than their ad libitum-fed littermates. This is complemented by data indicating that neuropeptide Y receptor Y2 (npy2r) deficiency reduces anxiety and increases food intake [100]. Neuropeptide Y (NPY) has powerful stimulatory effects on food intake. Anorexia may arise from pathological positive feedback loops: voluntary food restriction activates SIRT1, promoting anxiety, hyperactivity, and addiction to starvation, exacerbating dieting and exercising, thus further activating SIRT1 [101]. RES, as a SIRT1 agonist, can provoke anxiety-like behavior via restriction of food intake.

Numerous studies consider stress a trigger of anxiety-like behavior, categorized into three main groups. The first group considers acute stress as a provoking factor [102]. The second group focuses on the significance of chronic stress in developing anxiety-like behavior [103]. The third group examines anxiety-like behavior as a consequence of PTSD [104]. Notably, animal stress models often reproduce anxiety and depression disorders simultaneously.

In general, the cellular and molecular mechanisms of stress-related anxiety disorders share common features with other psychiatric diseases, especially depression. Psychiatric and neurodegenerative diseases are characterized by disorders of neuroplasticity, mitochondrial dysfunction, oxidative stress, and neuroinflammation. Specific aspects of the pathogenesis of anxiety disorders may be identified by synaptic interneuronal connections and the dominant brain regions where neuron circuits are disrupted. Therefore, nonspecific cytoprotectors like RES, which have demonstrated effectiveness in a wide range of neurological and psychiatric diseases, are also effective against stress-induced anxiety disorders. This is particularly relevant considering that SIRT1, the main target of RES, is implicated in stress–anxiety disorders.

It is generally accepted that anxiety disorders develop due to disturbances in interactions between monoamine neurotransmitters, namely, norepinephrine (NA), dopamine (DA), and serotonin (5-HT). 5-HT, NA, and DA are canonical neurons governing a range of biological activities, including sleep, alertness, eating, thermoregulation, pain, emotion, and memory, due to their broad projection distribution in distinct brain regions [105].

Anxiety in rodents is commonly studied using established laboratory tests such as the elevated plus maze (EPM) [106]. The EPM calculates the anxiety index (AI), an integral characteristic of anxiety-like behavior. The AI has been used to segregate stressed animals into low- and high-anxiety phenotypes. Recently, newer, less invasive methods of behavioral analysis, which rely on original ethological approaches, have gained traction. These methods monitor freely behaving rodents in their home-cage environment without intervention. In the home cage, stress exposures can provoke specific behavioral responses such as grooming, freezing, rearing, or surveying. Using this approach, it was shown that individual rodents exhibit either passive or active coping styles in response to the same stress exposures.

Active coping style (ACS) is characterized by “fight or flight” responses toward environmental threats, maximally expressing aggressiveness towards conspecifics. Conversely, a passive coping style (PCS) is characterized by avoidance of environmental threats and reduced aggression towards conspecifics [107]. ACS excludes the presence of anxiety, which signals danger and gradually turns into fear, whereas PCS provokes anxiety disorders. In studies involving predator stress (PS), behavior in the home cage was correlated with long-lasting consequences of stress exposures. ACS rats exhibited a low-anxiety phenotype, while PCS rats were associated with a high-anxiety phenotype. Notably, anxiety responses were tested 14 days poststress exposure, although behavior in the home cage was indicated immediately at the time of the stress cue [108].

Further research revealed that behavior in the home cage of stress-exposed rats, as well as the long-lasting consequences of PS, depended on the initial state of the animals. Using the hexobarbital sleep test (HST) to estimate the initial state of experimental animals allowed for the division of animals into fast and slow metabolizers (FM and SM, respectively) long before stress exposures [109]. It was found that FM rats were associated with ACS during stress exposures and exhibited low anxiety phenotypes poststress, while SM rats exhibited PCS and high anxiety phenotypes [109]. These results highlight the hereditary predisposition towards anxiety disorders.

Candidate genes for anxiety disorders have been identified, including genes related to monoaminergic neurotransmitter systems and hypothalamic–pituitary–adrenal (HPA) axis function. Among the most frequently studied candidate genes are the 5HTTLPR polymorphism of SLC6A4, the Val158Met polymorphism (rs4680) of catechol-O-methyltransferase (COMT), a promoter length polymorphism of monoamine oxidase A (MAO-A), and an RGS2 variant (rs4606) [110]. Notably, significant differences between FM and SM rats were observed in the expression of genes MAO-A and COMT, as well as in corticosterone metabolism in tissues [111].

Although the pathogenesis of stress-related anxiety disorders involves a complex tapestry of biological, environmental, and psychological factors, the primary pathway is intricately linked with abnormalities in neuronal circuits across various brain areas. In this context, stress and anxiety are part of a kaleidoscopic mosaic with common underpinnings and bidirectional links. Stressful events and anxious behavior act on similar specific brain regions, neurotransmitters, and a single neuro-endocrine axis and orchestrate homologous alterations in neuroplasticity.

The LHPA (limbic–hypothalamic–pituitary–adrenal) axis is certainly a central neuro-endocrine effector in the labyrinthine pathogenesis of stress-related anxiety disorders [112]. Glucocorticoids, the key hormones of the LHPA axis, can delve into the regulation of neuronal circuits. Mitochondrial dysfunction and neuroinflammation predetermine the severity of stress and anxiety disorders, creating an intricate interplay that transcends local disturbances within different sets of the brain.

Moreover, abnormalities in the interactions between the brain and other inner organs also contribute to the development of anxiety. This is especially justified in the context of the gut–brain, heart–brain, and liver–brain axes. These axes intertwine in a captivating manner, revealing that anxiety is not merely a local brain issue but part of a verdant and intricate systemic interaction. This reimagined perspective on stress and anxiety disorders highlights the enigmatic and multifaceted nature of these conditions, encouraging a more holistic approach to their study and treatment.

**Figure 5:**
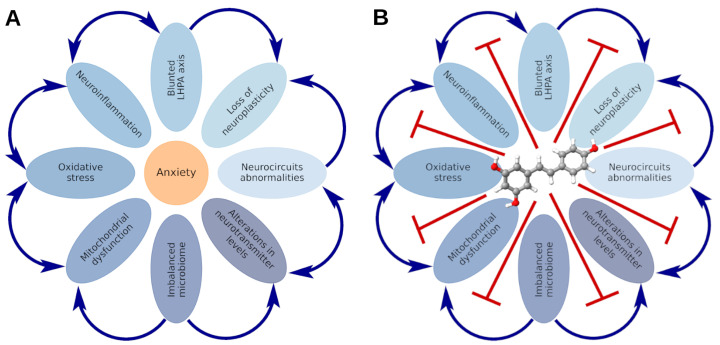
Depiction of the pathogenesis of stress-related anxiety disorders, focusing on neuroinflammation, oxidative stress, mitochondrial dysfunction, and other key factors (**A**). Illustration of resveratrol’s potential to address these factors, highlighting its therapeutic role in anxiety management (**B**).

At the systemic level, stress-related anxiety is characterized by dysregulation of the LHPA axis, disrupted interorgan interactions, imbalances in the microbiome, and impaired neuronal circuits. Neuroinflammation, mitochondrial dysfunction, and oxidative stress are key contributors to neuronal damage in the context of stress-related anxiety. Furthermore, stress-related anxiety leads to impaired neuroplasticity, which in turn results in synaptic dysfunction. Given the neuroprotective effects of RES on neuroplasticity, oxidative stress, and mitochondrial function, its use is well-justified for the treatment and management of anxiety disorders.

(Figure 5A) illustrates the key components of the pathogenesis of stress-related anxiety disorders, including neuroinflammation, oxidative stress, mitochondrial dysfunction, reduced neuroplasticity, abnormalities in neuronal circuits and neurotransmitter levels, aberrations in cerebral blood flow, impaired LHPA axis function, and abnormalities in the gut–brain and liver–brain axes. (Figure 5B) demonstrates the ability of resveratrol to correct each of these pathogenic factors, supporting its consideration as a therapy based on pathogenesis. The subsequent discussion highlights the role of the aforementioned factors in the development of anxiety and the significance of resveratrol in their correction.

## 4. Abnormalities in Neuronal Circuits and Neurotransmitter Levels in the Pathogenesis of Stress-Related Anxiety Disorders and the Efficacy of RES in Their Correction

The neural circuits promoting anxiety behavior are closely linked to stress experiences in both healthy and pathological conditions. Disruptions in brain connectivity contributing to anxiety may underlie stress-related disorders, including PTSD. Humans and animals share similar neural circuitry for anxiety behaviors [113]. Advances in optogenetics and electrophysiology have enabled the reconstruction of neural activities leading to anxiety-like behavior [114].

Stress-induced anxiety primarily involves the amygdala, prefrontal cortex, hippocampus, and striatum, though the hypothalamus, midbrain, and medulla oblongata are also implicated [115]. The amygdala plays a crucial role in detecting threats, fear learning, and memory for emotional events, making it central to anxiety-like disorders [116]. For example, PTSD studies highlight the role of glutamate excitotoxicity in amygdala hyperarousal [117].

Different amygdala subregions, including the basolateral amygdala (BLA) and central amygdala (CeA), engage in various circuits with regions like the hypothalamic paraventricular nucleus (PVN), nucleus accumbens, and bed nucleus of the stria terminalis (BNST) [118]. The hippocampus and PFC also play significant roles in anxiety behavior [119]. The PFC, in particular, supervises emotional states through its connections with the hippocampus and amygdala [118,119,120].

Dysregulation of the amygdala–hippocampus axis is linked to stress-related anxiety, with hippocampal neurogenesis being stimulated by antianxiety treatments such as electroshock therapy and corticotropin-releasing hormone (CRH)-1 antagonists [121]. The hippocampus responds to input from the amygdala and glucocorticoids, promoting anxiety under stress. Activation of BLA projections to the ventral hippocampus (vHPC) induces anxiogenic effects, while inhibition has anxiolytic effects [121]. Chronic stress alters neuronal activity, disrupts spinogenesis, and decreases spine stability [121]. These stress effects are reversed by RES treatment.

Neurotransmitters such as gamma-aminobutyric acid (GABA), glutamate, and monoamines (serotonin, norepinephrine, dopamine), along with neuropeptides like NPY and corticotropin-releasing factor (CRF), regulate anxiety within this network [122]. CRF acts as a neurotransmitter in the amygdala, and RES has been shown to influence levels of these neurotransmitters.

RES has been shown to to reduce CRF protein levels in the amygdala, leading to a reduction in anxiety-like behavior [123]. Furthermore, RES has been found to significantly suppress glutamate-induced currents in postsynaptic CA1 pyramidal neurons, with kainate and NMDA receptors being more sensitive to RES compared to AMPA receptors [124]. This inhibition of postsynaptic glutamate receptors likely works in conjunction with RES’s antioxidant properties to mitigate brain ischemic injury [125]. Molecular docking studies have also elucidated the interaction of RES with GABA aminotransferase, GABA receptors, and GABA-A transporter type 1, suggesting that RES’s anxiolytic effects may involve inhibiting the GABA reuptake transporter 1 protein, thereby increasing synaptic levels of GABA neurotransmitter [126].

RES treatment has been shown to enhance 5-HT levels in the pineal gland, hippocampus, and striatum, as well as increase levels of NA in the hippocampus and DA in the striatum. These effects are attributed to increased activity of enzymes such as tryptophan hydroxylase (in the pineal gland) and tryptophan hydroxylase-2 and tyrosine hydroxylase (in the hippocampus and striatum) [127]. Moreover, these hippocampal effects are correlated with RES-induced improvements in working memory [127]. Acute RES administration has been found to enhance cocaine-induced dopamine neurotransmission and behavioral responses, possibly by inhibiting dopamine catabolism via MAO-A and MAO-B [128].

In a mouse model of anxiety and depression behavior induced by social isolation with chronic unpredictable stress, RES treatment significantly increased levels of the neurotransmitters dopamine and serotonin in the prefrontal cortex, along with increased expression of NPY in the brain [129,130]. The NPY system plays a crucial role in mediating resilience to the harmful effects of stress, particularly in conditions such as PTSD [131].

RES has been shown to increase the release of calcitonin gene-related peptide (CGRP) from dorsal root ganglion (DRG) neurons isolated from wild-type (WT) mice. Moreover, significant increases in tissue levels of CGRP, insulin-like growth factor-I (IGF-I), and IGF-I mRNA, along with the immunohistochemical expression of IGF-I, were observed in the hippocampus three weeks after oral administration of resveratrol in WT mice. This enhancement was associated with significant improvements in angiogenesis and neurogenesis in the dentate gyrus of the hippocampus, as well as improvement in spatial learning in the Morris water maze test [132].

Currently, multiple effects of RES on dopaminergic, serotoninergic, and noradrenergic neurons, as well as on GABAergic and glutamatergic neurons, have been identified. These neuron types play crucial roles in the neurocirculation between brain regions such as the PFC, hippocampus, amygdala, striatum, hypothalamus, and monoaminergic nuclei of the midbrain. Furthermore, a glycosylated derivative of RES, 2,3,4^′^,5-tetrahydroxystilbene-2-*O*-β-d-glucoside (TSG), has been identified as a more effective option for enhancing long-term potentiation (LTP) in the hippocampus under physiological and pathological conditions compared to resveratrol alone. TSG, along with its parent molecule RES, can induce early LTP and restore fast excitatory postsynaptic potentials (EPSPs) in the hippocampus. Studies using various modalities, including pre- and post-whole-cell patch clamping techniques in the calyx of Held, have demonstrated that TSG, unlike RES, primarily promotes NMDA-mediated EPSC via the PKCβ cascade [133]. Inhibition of excitatory synaptic transmission by RES in the rat hippocampus has also been reported [124]. However, data on the consequences of direct RES infusion in brain areas involved in neuronal circuits implicated in anxiety disorders are limited, with only a single case of intrahippocampal RES infusion and a single case of RES infusion in the NAc reported in the literature.

However, further progress in research is hindered by the lack of data regarding the direct administration of RES into various brain structures. Unfortunately, there is also a lack of information regarding the influence of RES on the excitability of these neurons. The absence of such information limits our ability to fully evaluate the neuroprotective potential of RES. This gap underscores the importance of future research endeavors aimed at addressing these knowledge deficits.

The amygdala is a key driver of anxiety disorders, sending neural impulses to the prefrontal cortex, hippocampus, striatum, and other brain structures. The anxiolytic effects of RES in the context of stress are associated with its ability to reduce amygdalar excitability. Additionally, these effects are linked to an increase in serotonin levels in the hippocampus and prefrontal cortex, as well as dopamine levels in the striatum.

## 5. Aberrations in Cerebral Blood Flow in the Pathogenesis of Stress-Related Anxiety Disorders and the Efficacy of RES in Their Amelioration

Abnormalities in neuronal circuits linked to anxiety are often associated with reduced cerebral blood flow (CBF). Studies using the predator stress model revealed that high-anxiety rats displayed reduced basal CBF, endothelial dysfunction, and lower endothelial nitric oxide synthase (eNOS) mRNA levels, along with decreased brain dopamine levels [134]. Conversely, low-anxiety rats showed elevated carotid blood flow and increased dopamine levels, suggesting a negative correlation between anxiety and CBF [135]. This aligns with findings that glucocorticoid administration can reduce CBF [136]. Interestingly, lower anxiety in the predator stress model was linked to increased brain-derived neurotrophic factor expression and hippocampal peroxidation, indicating potential damage in this brain region.

It is conceivable that RES may reduce anxiety by enhancing CBF intensity. Human studies have shown that long-term daily intake of oral RES improves cerebral blood flow [137]. Furthermore, long-term administration of RES at low doses has been found to improve neurocognitive performance and modulate inflammatory pathways in the brain [138]. Investigating the contribution of RES to cerebral vasoprotective effects in the correction of anxiety disorders represents a promising new direction for research.

Disruption of cerebral blood flow is involved in the pathogenesis of anxiety disorders. Chronic stress is associated with alterations in cerebral circulation, which in turn correlate with reduced dopamine levels in the brain. RES has been shown to ameliorate disturbances in cerebral blood flow, highlighting its potential therapeutic benefit in managing these stress-induced abnormalities.

## 6. Neuronal Plasticity Abnormalities in the Pathogenesis of Stress-Related Anxiety Disorders and the Efficacy of Resveratrol in Correction

Psychosocial stress leads to atrophy of dendrites in the hippocampal CA3 region, with chronic stress reducing dendritic arborization in both the prefrontal cortex and hippocampus. This stress-induced anxiety is associated with persistent upregulation of BDNF in the basolateral amygdala [121]. Excessive glutamate inhibits BDNF expression through NMDA receptor activation, disrupting neuronal protection pathways like PI3K and ERK, thereby weakening the protective effect against excitotoxicity [139]. Stress also disrupts PFC inhibition of the amygdala, contributing to anxiety-like behavior [139].

Synaptic plasticity-related molecules such as BDNF, Arc, postsynaptic density protein 95 (PSD-95), and TrkB are crucial for maintaining synaptic function in stress-related anxiety [140]. Disrupted synaptic plasticity, particularly in the hippocampus, underlies susceptibility to social defeat stress and ethanol withdrawal-induced anxiety, associated with changes in BDNF, PSD-95, and NR2B levels [141]. Prenatal stress and ethanol exposure impair hippocampal neurogenesis, increase apoptosis, and reduce synaptic plasticity, leading to anxiety-like behaviors [142,143]. Ten-eleven translocation protein 3 (TET3), a DNA demethylation enzyme, links environmental stress with neuroplasticity and behavior. Inhibition of TET3 in the NAc enhances anxiety-like behavior by impairing dendritic spine density and decreasing the expression of synaptic plasticity genes like Bdnf while increasing immune-related genes [144]. Molecules that promote dendritic branching could serve as potential antianxiety treatments.

RES has been shown to diminish anxious behavior by upregulating BDNF levels in the hippocampus. Previous studies have reported that RES treatment ameliorates depressive behavior in chronic unpredictable mild stress (CUMS) rats by reducing LHPA axis hyperactivity and enhancing BDNF levels [145,146]. Overall, by upregulating BDNF and mediating synaptic function, RES facilitates interactions between neurotransmitters, which is especially important in stress-related behavioral disorders.

Anxiety-like behavior has been observed following toxicological intervention with $As_{2}O_{3}$, modulated by the estrogen-NMDAR-BDNF signaling pathway in the female mouse hippocampus [147]. Behavioral alterations and marker expression were restored in RES-supplemented mice. Anxiety behavior, along with decreased hippocampal BDNF, is also provoked by hyperalgesia, such as in the case of low back pain. RES treatment ameliorates anxious behavior and increases BDNF expression in the hippocampus of mice subjected to lumbar spine instability surgery [148].

In a chronic-acute combined stress (CACS) paradigm used to simulate irritable bowel syndrome (IBS) associated with behavioral complications such as anxiety, administration of RES before CACS for 3 weeks significantly reversed CACS-induced depression and anxiety-like behaviors and intestinal dysfunction in mice. This suggests a crucial role for *trans*-resveratrol in the treatment of IBS-like disorders. RES improved hippocampal neuronal remodeling and protected the ileal and colonic epithelial barrier structure against CACS insults [149]. Further study indicated that RES normalized phosphodiesterase 4A (PDE4A) expression and CREB/BDNF signaling, which were disturbed by CACS. Increased pCREB and BDNF expression were observed in the hippocampus after treatment with RES, while decreased pCREB and BDNF levels were found [150].

In mice subjected to chronic stress, RES treatment was accompanied by the alleviation of anxiety behavior and improved neuroplasticity. Chronic stress reduces SIRT1 activity in the dentate gyrus of the hippocampus, and pharmacologic and genetic inhibition of hippocampal SIRT1 function led to increased depression-like behaviors. Conversely, SIRT1 activation by RES blocked both the development of anxious behavior and aberrant dendritic structures elicited by chronic stress exposure. Furthermore, hippocampal SIRT1 activation increased the phosphorylation levels of ERK1/2 under stressed conditions. Viral-mediated activation and inhibition of hippocampal ERK2 led to antidepressive and prodepressive behaviors, respectively, [151].

The anxiolytic-like effect of RES treatment is mediated by phosphodiesterase 4 (PDE4), with a downstream increase in BDNF expression in the hippocampus. Neurobiological studies suggest that RES increases the phosphorylation of pCREB and BDNF levels in rats subjected to an animal model of PTSD.

Stress-related anxiety disorders are associated with disruptions in neuroplasticity, particularly in the hippocampus, which manifest as impaired synaptic signal transmission. The anxiolytic effects of resveratrol are linked to its ability to enhance the synthesis of BDNF in the hippocampus. RES promotes BDNF production in the hippocampus through the SIRT1 pathway, involving the participation of PDE4.

## 7. Peripheral Inflammation and Neuroinflammation in the Pathogenesis of Stress-Related Anxiety Disorders and the Efficacy of Resveratrol in Their Treatment

Peripheral inflammation in stress-related anxiety disorders is linked to imbalances between glucocorticoids (GCs) and pro-inflammatory cytokine signaling, with blunted GC signaling observed in PTSD patients [152,153,154]. Studies show increased levels of pro-inflammatory cytokines, such as interleukin-1 (IL-1), IL-6, and TNF-α, in PTSD, along with a possible reduction in anti-inflammatory cytokines like interleukin-4 (IL-4) and interleukin-10 (IL-10) [144,155]. Imbalances in immune cell compositions, including increased pro-inflammatory cells, have also been noted [156].

Neuroinflammation affects the brain by promoting excitotoxicity, disrupting glutamate transport, and altering cytokine levels via NMDA/AMPA and mGluR receptors [157]. It also impacts serotonin reuptake through TNF-α and IL-1β effects on serotonin transporter (SERT) [158]. Resveratrol (RES) antagonizes pro-inflammatory cytokines, downregulating SERT mRNA in stressed mice, and improves anxiety-like behavior [159]. RES also targets NF-κB pathways, ameliorating anxiety and depression-like behaviors in stress models and showing greater efficacy than fluoxetine in regulating NF-κB/NLRP3 signaling pathways [160,161]. RES was also beneficial in a mouse model of Lafora disease, improving anxiety-like behavior [162].

RES ameliorates LPS-induced anxiety-like behavior by attenuating Yes-associated protein (YAP)-mediated neuroinflammation and promoting hippocampal autophagy in mice. YAP, as a major downstream effector of the Hippo signaling pathway, plays a critical role in inflammation [163]. LPS treatment induced anxiety-like behavior, decreased sirtuin 1, and increased YAP expression in the hippocampus. Resveratrol attenuated LPS-induced anxiety-like behavior, an effect blocked by EX-527 (a sirtuin 1 inhibitor). Mechanistically, the anxiolytic effects of resveratrol were accompanied by a marked decrease in YAP, interleukin-1β, and ionized calcium binding adaptor molecule 1 (Iba-1) and a significant increase in autophagic protein expression in the hippocampus [163]. RES abrogates alcohol-induced anxious behavior by attenuating the inflammatory cascade in the adult rat brain. TNF-α, IL-1β, NF-κB, and caspase-3 levels in different brain regions (cerebral cortex and hippocampus) of ethanol-treated rats were downregulated by RES treatment. RES attenuated LPS-induced anxiety-like behavior. Mechanistically, the anxiolytic effects of RES were accompanied by a marked decrease in IL-1β and Iba-1, while there was a significant increase in autophagic protein expression in the hippocampus [163].

Neuroinflammation is a central mechanism in the pathogenesis of anxiety disorders. It is triggered by the activation of the NF-κB pathway, secretion of pro-inflammatory cytokines by glia, increased expression of YAP protein, and suppression of the SIRT1 pathway. Resveratrol mitigates neuroinflammation by activating the SIRT1 pathway and inhibiting YAP protein expression. The attenuation of neuroinflammation by resveratrol is accompanied by a reduction in pro-inflammatory cytokines.

## 8. Mitochondrial Dysfunction in the Pathogenesis of Stress-Related Anxiety Disorders and the Efficacy of RES in Their Correction

Dysfunctional mitochondrial dynamics can incite innate immune responses in resident and infiltrating cells, such as microglia, astrocytes, and oligodendrocytes, involving calcium-dependent immune activation, phosphorylation of transcription factors, and cytokine secretion [164,165].

The pivotal role of mitochondria in the evocation of anxiety disorders is illustrated by findings that mitochondrial transplantation improves anxiety- and depression-like behaviors in aged, stress-exposed rats [166]. Social status also predicts behavioral stress susceptibility and the metabolic profile in the NAc after chronic social defeat stress [167]. Hollis et al. reported that mitochondrial function in the NAc is crucial for social hierarchy establishment and is critically involved in the low social competitiveness associated with high anxiety [168]. Notably, the expression levels of glucocorticoid receptors (GRs) in the NAc of high-anxious, submissive-prone rats are lower than those of their low-anxious, dominant-prone counterparts [169]. Hence, the glucocorticoid receptor in the nucleus accumbens plays a crucial role in social rank attainment in rodents.

Mitochondria are vital organelles involved in the biological stress response, making them key to understanding stress-related illnesses like PTSD. Recent research highlights the link between anxiety and mitochondrial dysfunction, with significant changes in mitochondrial function observed in highly anxious individuals and those with mitochondrial disorders [170]. Experimental models confirm the role of mitochondrial dysfunction, including disrupted oxidative phosphorylation and metabolic pathways, in the development of anxious behavior [171].

Mitochondrial functions such as bioenergetics, oxidative stress, and apoptosis are closely tied to anxiety [172]. Dysfunction in the nucleus accumbens (NAc), a brain region crucial for motivation and reward, is particularly significant, as it affects neuroinflammation and immune responses involving microglia and astrocytes [165]. Mitochondrial transplantation has shown promise in alleviating anxiety and depression-like behaviors in stress-exposed rats [166]. Social status and GR expression in the NAc also play crucial roles in stress susceptibility and social hierarchy, linking low social competitiveness with high anxiety [168,169].

RES has a protective effect on spine plasticity and mitochondrial function in the nucleus accumbens of rats subjected to social isolation, accompanied by an improvement in anxiety-like behavior. Furthermore, RES increased the activity of cytochrome c oxidase (COX) and upregulated mRNA levels of COX5a, COX6a1, and COX7c [173]. It has been revealed that the influence of RES on the NAc is mediated by SIRT1. When resveratrol, a pharmacological activator of SIRT1, was directly infused bilaterally into the NAc, an increase in depression- and anxiety-like behaviors was observed. Conversely, intra-NAc infusions of EX-527, a SIRT1 antagonist, reduced these behaviors; EX-527 also reduced acute stress responses in stress-naive mice.

Moreover, SIRT1 levels were increased directly in the NAc by the use of viral-mediated gene transfer, which resulted in an increase in depressive- and anxiety-like behaviors when mice were assessed in the open-field, elevated plus maze, and forced swim tests. Using a Cre-inducible viral vector system to overexpress SIRT1 selectively in dopamine D1 or D2 subpopulations of medium spiny neurons (MSNs) in the NAc indicated that SIRT1 promotes depressive-like behaviors only when overexpressed in D1 MSNs, with no effect seen in D2 MSNs. Conversely, selective depletion of SIRT1 in the NAc using viral-Cre in floxed SIRT1 mice resulted in decreased depression- and anxiety-like behaviors. Together, these results demonstrate that SIRT1 plays an essential role in the NAc in regulating mood-related behavior [174].

In mice subjected to the CUMS paradigm, RES treatment reversed anxiety-like behavior and improved mitochondrial dysfunction via the SIRT1/PGC1α/SIRT3 pathway [175]. In another study using the adolescent social isolation stress (SIS) paradigm, the presence of anxious behavior, reduced NAc ATP levels, and reduced mitochondrial number in female rats were observed. These effects of SIS were reversed by RES treatment. In the SIS paradigm, a significant decrease in mitochondrial function-related genes, such as the COX-related genes, was revealed, whereas RES treatment increased the expression of COX genes (Cox5a, Cox6a1, and Cox7c) and the activity of COX [175].

In the social defeat paradigm in rats, increased $Ca^{2+}$ concentrations led to impaired mitochondrial membrane potential and the opening of the mitochondrial permeability transition pore. RES treatment prevented mitochondrial impairment [173].

Mitochondrial dysfunction is a key factor in the pathogenesis of anxiety disorders. The neuroprotective effect of RES on mitochondrial dysfunction is associated with its anxiolytic properties under stress.

## 9. Oxidative Stress: Implications in the Pathogenesis of Stress-Related Anxiety Disorders and the Therapeutic Potential of RES

**Figure 6:**
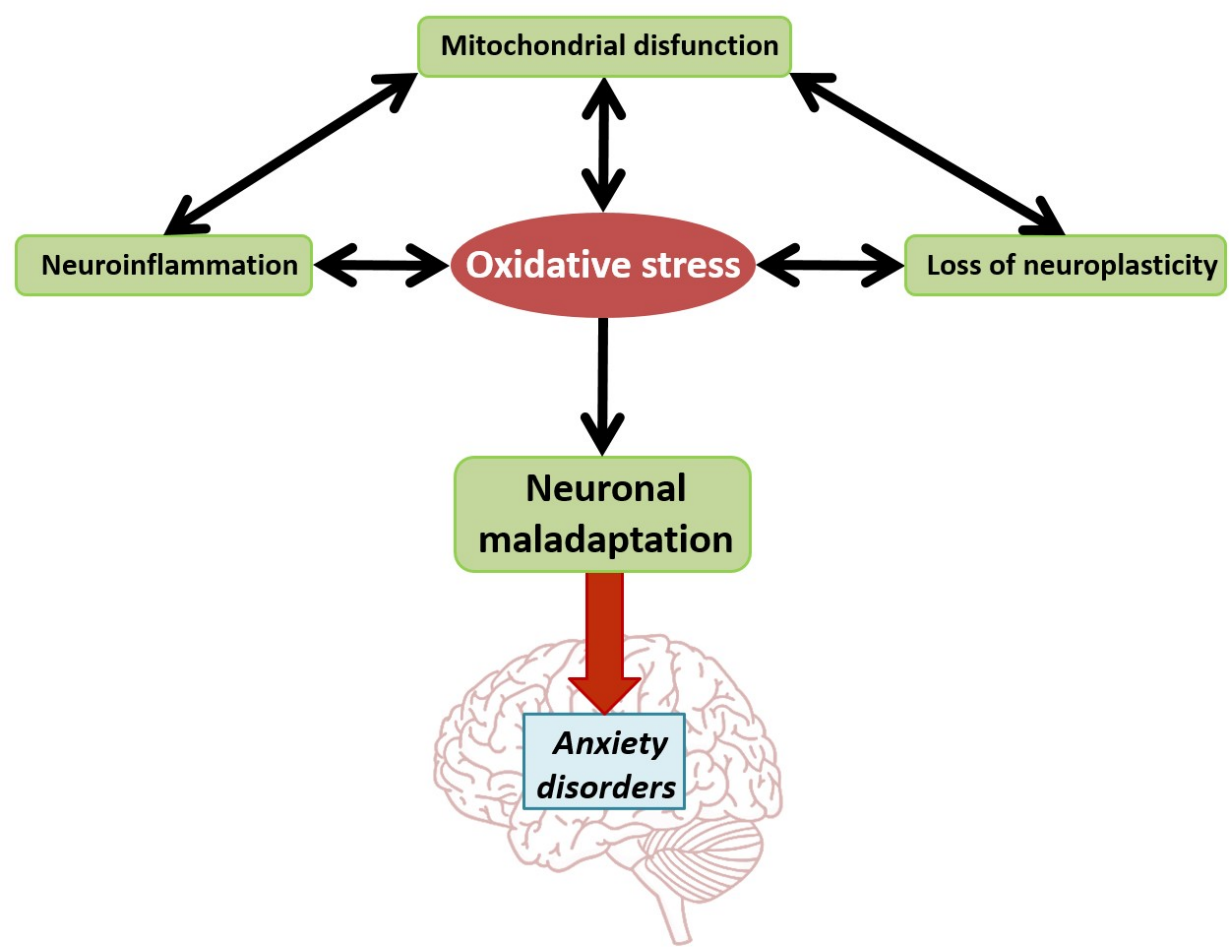
Oxidative stress in the brain is involved in the development of anxiety disorders.

Oxidative stress (OS) is central to neuronal abnormalities in stress-related anxiety and depression (Figure 6) [176]. Insufficient antioxidants can provoke OS, and silencing Nrf2, a key antioxidant regulator, leads to anxiety-like behavior [177,178]. Deletion of glutathione peroxidase (GSPX) in dopaminergic neurons and mitochondrial-targeted overexpression of catalase both impact anxiety behaviors [179,180]. OS is a consequence of mitochondrial dysfunction and neuroinflammation, with a bidirectional relationship between these factors.

Mitochondrial dysfunction linked to anxiety disorders triggers OS, affecting electron transport chain (ETC) complexes and leading to ATP deficiency and increased mtROS [181,182]. Excess ROS causes excitotoxicity through Ca^2+^ dysregulation and GLT-1 impairment. Monoamine oxidase (MAO) activation further exacerbates OS and mitochondrial dysfunction, reducing hippocampal 5-HT and BDNF levels in stressed rats [183,184].

ROS inhibits BDNF by elevating NF-κB activity, while BDNF provides antioxidant protection via TrkB and NF-κB signaling [185]. Synaptic dysfunction linked to OS disrupts neurotransmission, with lipid raft damage contributing to synaptosome impairment [186]. OS also exacerbates neuroinflammation through AP-1 and NF-κB pathways, enhancing inflammatory gene expression [187].

Abdel-Wahab and colleagues highlighted the link between resveratrol’s ability to improve anxious behavior induced by long-term intermittent hypoxia (IH) and its antioxidant properties [188]. Long-term IH induces memory deficits and hippocampal oxidative stress, characterized by increased thiobarbituric acid reactive substances (TBARS) and p47Phox expression, a subunit of NADPH oxidases. RES attenuates stress-induced anxiety and spatial memory deficits in a dose-dependent manner, as demonstrated by elevated plus maze and Morris water maze tests in IH-exposed animals. Furthermore, RES stimulates apurinic/apyrimidinic endonuclease 1 (APE1), a multifunctional protein involved in DNA repair and cell survival after exposure to cytotoxic agents [188]. Treatment with RES also increases glutathione levels and glutathione peroxidase activity while simultaneously reducing TBARS levels in the hippocampus of IH-exposed young rats [188].

Several studies investigating RES’s antioxidant potential in experimental stress-related anxiety paradigms have shown promising results, with its antioxidant effects aligning with its ability to mitigate neuroinflammation, mitochondrial dysfunction, and apoptosis and stimulate autophagy, particularly in the hippocampus [189]. In rat pups subjected to a maternal deprivation paradigm, RES treatment reversed abnormalities in anxious behavior and hippocampal lipid peroxidation while also antagonizing alterations in monoamine levels [190]. Notably, oxidative stress and changes in monoamine concentration are closely linked in this context, likely influenced by mitochondrial impairment associated with anxious behavior.

In anxiety disorders, oxidative stress triggers disruptions in neuroplasticity and contributes to the development of neuroinflammation, mitochondrial dysfunction, and impaired neurotransmission. RES mitigates oxidative stress in brain neurons through its antioxidant properties, providing protection against these detrimental effects. Mitochondrial dysfunction remains a critical factor in the pathogenesis of anxiety disorders.

## 10. Blunted LHPA Axis Function in the Pathogenesis of Stress-Related Anxiety Disorders and the Efficacy of RES in Their Correction

Anxiety-like behavior under stress is linked to dysregulation of the limbic–hypothalamic–pituitary–adrenal (LHPA) axis [191]. The limbic system, including the hippocampus, amygdala, and prefrontal cortex, is rich in glucocorticoid receptors (GRs) [192]. In the hippocampus, glucocorticoids also act via membrane-associated mineralocorticoid receptors (MRs) [192]. GR-GC complexes translocate to mitochondria, enhancing mitochondrial oxidation and ROS production [193]. Glucocorticoids recruit histone deacetylase enzymes to reduce inflammation, similar to SIRT effects [194]. SIRT1-mediated deacetylation of methyl CpG binding protein 2 (MeCP2) contributes to BDNF expression [195].

Glucocorticoids increase glutamate excitability in various brain regions, evidenced by the lack of extracellular glutamate elevation in adrenalectomized rats. In the amygdala, glutamate enhances BDNF/TrkB pathway activation, while in the hippocampus, it decreases BDNF expression and spine numbers. The biphasic actions of glucocorticoids are time-dependent [196].

The interaction between growth factors like BDNF and glucocorticoids is crucial for understanding anxiety disorders [141]. The LHPA axis influences ROS generation, mitochondrial gene expression, and metabolism through GRs, and it modulates neuroinflammation via cytokines and GR/NF-κB pathways [197]. GR activation reduces BDNF, while BDNF influences GR phosphorylation and the glucocorticoid transcriptome [198]. HPA hormones also affect gut microbiota and intestinal permeability, linking the microbiota–gut–brain axis to the HPA [199].

Glucocorticoid metabolism involves 11β-hydroxysteroid dehydrogenases (11βHSD1 and 11βHSD2), regulating the active/inactive forms of GCs [200]. A mathematical model in PTSD research highlights hepatic 11βHSD1 as a key factor in plasma corticosterone dynamics, which affects brain MAO-A activity and noradrenaline levels [201].

Recent studies indicate that RES targets the LHPA. RES protects neurons against PTSD-like stress insults by regulating LHPA axis function and activating downstream neuroprotective molecules such as protein kinase A (PKA), pCREB, and BDNF expression [202]. Furthermore, RES reverses the reduction of GRs in the cingulate cortex of mice subjected to single prolonged stress paradigms. RES acts in a dose-dependent manner to reverse anxiety-like behavior induced by PTSD-like stress but does not significantly affect naive animals. These findings indicate that RES mitigates neurological deficits caused by traumatic stress without affecting normal conditions.

In anxiety disorders, significant alterations in glucocorticoid levels occur, leading to the remodeling of glucocorticoid-dependent signaling pathways in limbic brain structures. Stress-induced anxiety is associated with increased activity of 11βHSD1, which directly impacts glucocorticoid levels. The anxiolytic effects of RES are linked to its ability to reduce 11βHSD1 activity, prevent the reduction of GR, and enhance the expression of PKA and pCREB.

## 11. Abnormalities in the Gut–Brain and Liver–Brain Axes in the Pathogenesis of Stress-Related Anxiety Disorders and the Efficacy of Resveratrol in Their Correction

The gut-brain axis, also known as the microbiota-gut-brain axis, is a complex signaling system between the brain and the gastrointestinal (GI) tract. It involves signaling molecules, gut hormones, and immune mediators from the gut microbiota [203]. Research into gut-derived signaling molecules like serotonin, epinephrine, and norepinephrine has highlighted their role in gastrointestinal dysbiosis and anxiety disorders [204]. This axis is crucial in regulating intestinal physiology.

Psychological stress activates the hypothalamic-pituitary-adrenal axis, impacting intestinal barrier function, gut microbiota composition, and behavior [205]. Meta-analyses have shown altered gut microbiota diversity and composition in PTSD patients, potentially linking specific bacterial taxa to these changes [206]. Traumatic stress disrupts gut barrier functions, increasing the risk of comorbidities in PTSD [207].

Liver transplantation patients often face neuropsychiatric disorders like depression and anxiety, which can impair rehabilitation and quality of life [208]. Post-transplant gut microbiota changes affect mental health, contributing to depression and anxiety [208].

Studies show that gut microbiome alterations affect anxiety behavior in animal models. Rats in the learned helplessness paradigm and mice exposed to chronic social defeat stress exhibited significant changes in microbial diversity, such as increased *Bacteroides* spp. [209]. Germ-free mice showed less anxiety and more motor activity, with elevated neurotransmitters NA, DA, and 5-HT [210]. Additionally, germ-free mice displayed increased depression-like behaviors after receiving fecal microbiota from patients with major depressive disorder (MDD) [211]. The microbiota may increase blood–brain barrier permeability, promoting anxious behavior during stress.

RES has been shown to induce anxiolytic-like behavior in stress-related IBS by regulating gut–brain interactions [212]. Specifically, resveratrol significantly increased PKA, phosphorylated cAMP-response element binding protein (p-CREB), and BDNF expression in the hippocampus of IBS rats while decreasing PKA, p-CREB, and BDNF levels in the ileum and colon [212]. Notably, RES is a target of gut microbiota. Microbial conversion of RES occurs through three pathways: one leading to the production and excretion of dihydroresveratrol, another producing lunularin, and a third resulting in the equivalent production of both catabolites. Lunularin, also known as 3,4^′^-dihydroxybibenzyl, is a *trans*-resveratrol catabolite obtained by its reduction to dihydroresveratrol and subsequent dehydroxylation at the 5-position. Almost all lunularin producers also excrete 3,4^′^-dihydroxy-*trans*-stilbene [213].

Recent studies have reported the efficacy of RES treatment in the predator scent stress paradigm, another PTSD model [214]. However, RES treatment has indicated two phenotypes among PTSD rats: treatment-sensitive rats (TSRs) and treatment-resistant rats (TRRs). In TSRs, post-treatment, RES ameliorated anxiety-like behavior and reversed plasma corticosterone concentration abnormalities. In contrast, in TRRs, RES treatment aggravated anxiety-like behavior and decreased plasma corticosterone concentration. In TSRs, hepatic 11βHSD1 activity was suppressed, with a concomitant increase in CYP3A activity. In TRRs, the activities of both enzymes were suppressed. Thus, the resistance of PTSD rats to RES treatment is associated with abnormalities in the hepatic metabolism of glucocorticoids. Notably, an 11βHSD1 inhibitor is capable of inducing the AMPK/SIRT1 signaling pathway, which protects against PTSD [215]. Meanwhile, RES can also mechanically suppress 11βHSD1 activity in the liver. In silico studies have revealed hydrogen bonds between the hydroxyl group of RES and Thr124 in 11βHSD1, as well as hydrophobic interactions responsible for the binding. Evaluating the impact of RES treatment on 11βHSD1 activity is especially significant given the high correlation (greater than 0.9) between the anxiety index and the activity of this enzyme in PTSD rats [216]. This finding aligns with data linking hepatic 11βHSD1 and brain MAO-A activities in PTSD rats.

Dysregulation of the microbiota is implicated in the pathogenesis of anxiety disorders. Additionally, disruptions in the liver–brain axis also contribute to the development of anxiety disorders under stress. The anxiolytic effects of RES are associated with its ability to correct both stress-induced microbiota disturbances and disruptions in the liver–brain axis.

## 12. SIRT1-Dependent Pathways and the Dual Effect of Resveratrol on Anxiety Behavior

Increasing evidence suggests that epigenetic alterations, such as histone modifications and DNA methylation, underlie the neuroprotective effects of RES in anxiety disorders [217]. Enzymes like acetylases and methylases are crucial in these processes. The amygdala, a key brain region for anxiety, shows increased SIRT1 levels in response to caloric restriction, implicating this sirtuin in anxiety modulation.

Polymorphisms in the SIRT1 gene, such as SNPs (rs10997870, rs12778366), are associated with anxiety disorders, panic disorder, and social phobia [218]. Brain-specific SIRT1 knockout mice exhibit reduced anxiety, while global SIRT1 overexpression increases anxiety and depression susceptibility [150]. A high-fat diet can induce anxiolytic-like behaviors via SIRT1 regulation [219]. Pharmacological inhibition of SIRT1 in the NAc reduces anxiety- and depression-like behaviors [174]. Stress-induced SIRT1 expression in the NAc regulates anxiety behaviors, and SIRT1 antagonists reduce these behaviors [174].

**Figure 7:**
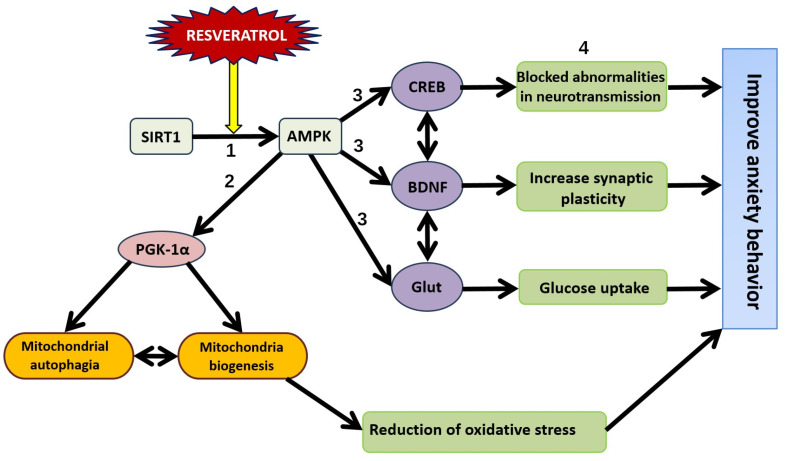
The main molecular pathways supporting the antianxiety effects of resveratrol include the SIRT1/AMPK/CREB pathway: RES reverses the imbalance in neurotransmitters (1); the SIRT1/AMPK/BDNF pathway: RES improves synaptic plasticity (2); the PGK-1α pathway: RES increases the biogenesis of mitochondria (3); and the AMPK/Glut pathway: RES improves glucose uptake by neuronal cells (4).

In a chronic stress mouse model, anxious behavior is linked to decreased SIRT1 in the prefrontal cortex and increased levels in the hippocampus (Figure 7) [220]. Overexpression of *N*-acetyltransferase in the hippocampus correlates with anxiety-like behavior and higher SIRT1 levels, but conflicting reports exist regarding hippocampal SIRT1 expression. In the CUMS paradigm, decreased SIRT1, increased pro-inflammatory cytokines, and altered neurotransmitter levels are observed, with Jiaotai pills improving these abnormalities potentially by upregulating SIRT1 [221].

Apelin-13 promotes neuroprotection and anxiolytic effects through increased SIRT1 acetylated p65 (lysine 310) in CNH-treated mice [222]. Dysregulation of the apelin-SIRT1-NF-κB axis may lead to neuroinflammation and anxiety-like behavior. LPS-induced anxiety is linked to reduced SIRT1 and increased IL-1 [222].

Metformin, an AMPK activator, reduces anxiety-like behavior postnicotine withdrawal through an AMPKα-dependent mechanism in the hippocampus [223].

Dysfunction in the SIRT1-PGC-1α mitochondrial pathway is associated with fear generalization and anxiety-like behavior triggered by SPS [224]. The Anshen Dingzhi prescription, from the Qing Dynasty’s “Yi Xue Xin Wu” (1732 CE), improves anxiety by activating the SIRT1-PGC-1α pathway, mitigating mitochondrial dysfunction [224]. Neuronal SIRT1 interacts with GR in a glucocorticoid-dependent manner, indicating its involvement in glucocorticoid signaling pathways in behavioral disorders [225].

Glucocorticoid signaling in the brain increases MAO-A mRNA levels, with SIRT1/NHLH2/ MAO-A pathways driving anxiety-like behavior. SIRT1 influences mood by deacetylating NHLH2, which activates MAO-A transcription. In SIRT1-overexpressing mice, MAO-A protein levels are high, and SIRT1’s activity modulates MAO-A via NHLH2. Specifically, NHLH2 is hypoacetylated in SIRT1 OX mice compared to wild-type, highlighting SIRT1’s role in anxiety [226].

Li et al. used genetic and pharmacological methods to target SIRT1 and investigate its effects on SPS-induced PTSD-like behaviors [227]. EX527, a SIRT1 inhibitor, was administered to the ventral CA1 of the hippocampus, preventing increased SIRT1 activity in SPS-exposed mice. EX527 rescued NHLH2 acetylation and inhibited MAO-A expression in the vCA1, reducing serotonin decomposition into 5-hydroxyindoleacetic acid (5-HIAA). Golgi staining showed improved neuronal plasticity, with fewer dendritic spines and reversed dendritic atrophy. EX527 also alleviated fear conditioning and anxiety-like behaviors, linking PTSD-like symptoms to increased SIRT1 activity in the CA1 area.

**Figure 8:**
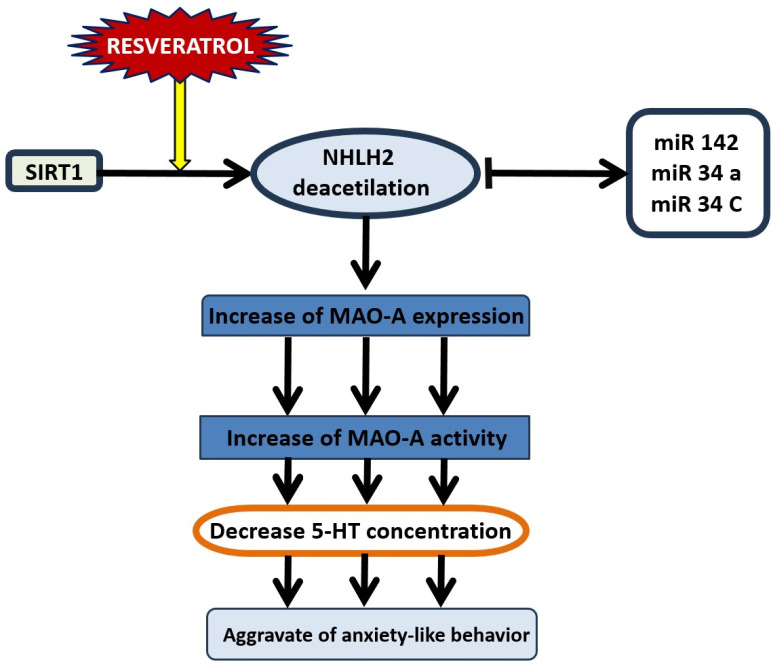
Resveratrol aggravates anxiety disorder via the SIRT1/NHL2/MAO-A pathway. RES exhibits a dual effect on stress-related anxiety behavior; while it has antianxiogenic properties, it can also mediate proanxiogenic effects through SIRT1 activation. SIRT1 induces the deacetylation of NHLH2 transcriptional factors, leading to increased MAO-A expression and decreased 5-HT concentration in neurons, which enhances anxiety behavior. The miRNAs miR-142, miR-34a, and miR-34c downregulate the proanxiety effect of the SIRT1/NHLH2/MAO-A pathway.

RES, as a SIRT1 activator, exhibits behavioral effects similar to the clinical antidepressant fluoxetine [228]. SIRT1’s influence on behavior may be mediated by changes in serotonin and other neurotransmitter levels affecting anxiety. SIRT1 OX mice have lower levels of 5-HT and higher levels of 5-HIAA compared to brain-specific SIRT1 knockout (OX) mice. Additionally, SIRT1 OX mice exhibit reduced levels of DA and NA, indicating elevated MAO-A activity in noradrenergic, serotonergic, and dopaminergic neurons. The SIRT1/NHLH2/MAO-A pathway is downregulated by miR-142, miR-34a, and miR-34c, with RES acting as an inhibitor of miR-142 (Figure 8) [229]. Notably, SPS rats show increased microRNA-142-5p levels in the amygdala and a concurrent reduction in Npas4, an activity-regulated transcription factor implicated in stress-related psychopathologies.

Considering SIRT1 as the primary target of RES, there is a hypothesis that RES could potentiate anxious behavior, especially in PTSD. However, reports on the effects of RES in PTSD suggest the opposite. RES has been shown to ameliorate anxiety-like behaviors and fear memory deficits in a rat model of post-traumatic stress disorder.

In studies, RES at doses of 10, 20, and 40 mg/kg (administered via gavage) reversed TDS-induced decreases in the percentage of time spent in the center of the arena as well as the number of open-arm entries and the time spent in open arms in the open-field and elevated plus maze tests. It also decreased the percentage of freezing time in the contextual fear paradigm, which was increased in TDS-treated rats [230].

Further studies indicated that TDS-induced abnormalities in the LHPA axis were reversed by RES. It reversed the increased adrenal gland index and corticotropin-releasing factor levels and rescued the differential expression of the GR in the hypothalamus, hippocampus, and amygdala. Neurobiological studies suggested that RES increased the p-CREB and BDNF levels, which were decreased in rats subjected to TDS.

These results provide compelling evidence that RES protects neurons against PTSD-like stress insults by regulating LHPA axis function and activating downstream neuroprotective molecules such as p-CREB and BDNF expression [230].

RES supplementation in the diet of PS-subjected rats has been shown to segregate the stressed rats into sensitive and resistant phenotypes to this polyphenol intervention [231]. This observation supports the notion that RES exerts a dual impact on anxiety behavior. Notably, both antagonistic effects appear to be mediated initially by SIRT1 and ultimately by MAO-A. Improved anxiety behavior is associated with decreased MAO-A gene expression and enzymatic activity, whereas aggravated anxiety corresponds with increased MAO-A activity. This dual impact is consistent with data indicating that RES suppresses MAO activity both in vitro and in vivo [232,233].

Interestingly, decreased MAO activity might also be associated with anxious behavior. Endogenous inhibitors of MAO-A and MAO-B, known as tribulins, can induce anxiety under stress conditions [234]. Therefore, both excessive and insufficient MAO-A activity can lead to increased anxiety, whereas moderate changes in MAO-A activity can improve anxious behavior.

To understand the behavioral effects of MAO-A, it is essential to consider its correlation with changes in the levels of monoamines, their receptors and transporters, and particularly of COMT, another enzyme involved in the metabolism of dopamine and norepinephrine. Although COMT does not directly impact serotonin metabolism, its role in dopamine and norepinephrine regulation is significant.

Apart from its effects on MAO-A, RES may improve anxiety behavior by influencing other targets within monoamine signaling pathways. Studies have shown that limited cheese intake supplemented with RES improves anxiety behavior in rats subjected to the predator tress paradigm. This improvement is associated with increased gene expressions of MAO-A, DAT, and BDNF in the hippocampus. In this anxiety model, the increased expression of BDNF reflects the neuroprotective properties of resveratrol.

In contrast, PS alone increases anxiety behavior by decreasing MAO-A gene expression and increasing COMT gene expression, leading to reduced dopamine concentration in the hippocampus. Cheese supplementation with RES resulted in decreased 5-HT turnover, consistent with reports of RES downregulating the SERT, similar to the effects of selective serotonin reuptake inhibitors (SSRIs). This suggests that RES may target SERT independent of SIRT1 [235].

For improved anxiety behavior, enhancing synaptic plasticity is also desirable. Stable neurotransmission is crucial for resilience to behavioral disorders. The neuroprotective effect of RES via the SIRT1/AMPK/BDNF pathway contributes significantly to improved anxiety behavior.

RES, through its interaction with SIRT1, can exert both anxiolytic and anxiogenic effects. The anxiolytic effects of resveratrol are mediated via the SIRT1/AMPK/CREB pathway. Conversely, its anxiogenic effects are associated with the SIRT1/NHL2/MAO-A pathway.

## 13. Conclusions

The neuroprotective effects of RES improve stress-related behavior by mitigating neuronal damage, neuroinflammation, mitochondrial dysfunction, and loss of neuroplasticity, all of which are implicated in the pathogenesis of stress-related anxiety. RES primarily acts through SIRT1 and AMPK to correct these deleterious consequences of stressful events in the brain. However, there is a scarcity of studies employing SIRT1 and AMPK inhibitors to analyze the mechanisms of RES’s neuroprotective action. Nevertheless, in silico data demonstrate RES’s ability to directly bind to other proteins involved in mediating its neuroprotective and anxiolytic effects. For instance, our data suggest that 11βHSD1 has binding sites for RES. It would be valuable to evaluate in silico the potential of RES to directly target neurotransmitter receptors and their transport proteins, bypassing SIRT and AMPK.

Interestingly, RES exhibits dual effects on anxious behavior. While most studies report anxiolytic effects of RES under conditions of stress, anecdotal evidence suggests that RES may enhance stress-related anxious behavior. Future studies will need to determine the extent to which RES’s effects on anxious behavior are influenced by the animal’s initial state and coping strategy. The hexobarbital test could serve as a useful tool for these investigations. Additionally, it will be necessary to assess how RES’s effects differ between fast and slow metabolizers, considering that susceptibility to anxious behavior is typical only for slow metabolizers. RES may be as effective as selective serotonin reuptake inhibitors in correcting anxiety disorders. However, the clinical use of RES is limited by its rapid metabolism via cytochrome P450 enzymes. Therefore, an auspicious direction for research is the development of metabolically stable forms of RES, as well as functional products enriched with RES.

## Figures and Tables

**Table 1:** The neuroprotective effects of RES treatment (in vitro data).

Organism Neuronal Culture	Molecular Target	Effects of RES	References
PC 12 cells	p53	RES prevents the proapoptotic increase in nuclear p53 induced by high glucose levels.	[19]
N9 microglial cells	NF-κB	RES reduces apoptotic neuronal cell death induced by neuroinflammation.	[20]
primary cultured neurons	SIRT1, SIRT3, PGC1α	RES may ameliorate manganese (Mn)-induced neuronal injury and mitochondrial dysfunction in primary cultured neurons by activating the SIRT1/PGC-1α signaling pathway, with SIRT3 being essential for promoting mitochondrial biogenesis and attenuating Mn-induced mitochondrial dysfunction.	[21]
H19-7 hippocampal neuronal cells	superoxide dismutase, catalase, glutathione reductase	RES treatment attenuated the accumulation of lipid peroxide levels, upregulated antioxidant activities, and improved the expression of memory-associated proteins in Aβ-treated H19-7 cells.	[22]
primary cultures of rat cortical neurons	Nrf-2	RES treatment at various time points increased neuronal viability and inhibited neuronal apoptosis in vitro, at least in part, by enhancing the activation of the Nrf-2 signaling pathway.	[23]
culture of dopaminergic neurons	intracellular free calcium, reactive oxygen species	RES enhances cell viability and reduces apoptosis by attenuating MA-induced reactive oxygen species (ROS) production and calcium overload. It protects dopaminergic neurons from cytotoxicity by inhibiting $Ca^{2+}$ and oxidative stress.	[24]
mice mesencephalic and cortical primary cultures	complex III of mitochondrial respiratory chain	A significant reduction in glutamate-induced radical formation was observed in cultures treated with resveratrol, demonstrating the antioxidant potential of RES.	[25]
rat cerebellar granule neurons (CGNs)	mitochondrial complex I, SIRT1	RES exhibits beneficial effects against mitochondrial dysfunction and prevents cell death in neuronal cells.	[26]
SH-SY5Y cells	AMPK	RES rescues SH-SY5Y cells from oxygen-glucose deprivation (OGD)-mediated mitochondrial deficiency and restores the transcript expression levels of PGC-1α and mitochondrial genes.	[27]

## Data Availability

No new data were created or analyzed in this study. Data sharing is not applicable to this article.

## References

[1] Prakash V., Bose C., Sunilkumar D., Cherian R.M., Thomas S.S., Nair B.G. (2024). Resveratrol as a Promising Nutraceutical: Implications in Gut Microbiota Modulation, Inflammatory Disorders, and Colorectal Cancer. Int. J. Mol. Sci..

[2] Rana A., Samtiya M., Dhewa T., Mishra V., Aluko R.E. (2022). Health benefits of polyphenols: A concise review. J. Food Biochem..

[3] Gambini J., Inglés M., Olaso G., Lopez-Grueso R., Bonet-Costa V., Gimeno-Mallench L., Mas-Bargues C., Abdelaziz K.M., Gomez-Cabrera M.C., Vina J. (2015). Properties of Resveratrol: In Vitro and In Vivo Studies about Metabolism, Bioavailability, and Biological Effects in Animal Models and Humans. Oxid. Med. Cell. Longev..

[4] Gugleva V., Zasheva S., Hristova M., Andonova V. (2020). Topical use of resveratrol: Technological aspects. Pharmacia.

[5] Chow H.H.S., Garland L.L., Hsu C.H., Vining D.R., Chew W.M., Miller J.A., Perloff M., Crowell J.A., Alberts D.S. (2010). Resveratrol Modulates Drug- and Carcinogen-Metabolizing Enzymes in a Healthy Volunteer Study. Cancer Prev. Res..

[6] VanElzakker M.B., Kathryn Dahlgren M., Caroline Davis F., Dubois S., Shin L.M. (2014). From Pavlov to PTSD: The extinction of conditioned fear in rodents, humans, and anxiety disorders. Neurobiol. Learn. Mem..

[7] Johnson L.R., McGuire J., Lazarus R., Palmer A.A. (2012). Pavlovian fear memory circuits and phenotype models of PTSD. Neuropharmacology.

[8] Tye K.M. (2018). Neural Circuit Motifs in Valence Processing. Neuron.

[9] Bhattacharya A., Chakraborty M., Chanda A., Alqahtani T., Kumer A., Dhara B., Chattopadhyay M. (2024). Neuroendocrine and cellular mechanisms in stress resilience: From hormonal influence in the CNS to mitochondrial dysfunction and oxidative stress. J. Cell. Mol. Med..

[10] Morella I.M., Brambilla R., Morè L. (2022). Emerging roles of brain metabolism in cognitive impairment and neuropsychiatric disorders. Neurosci. Biobehav. Rev..

[11] Kaplan G.B., Dadhi N.A., Whitaker C.S. (2023). Mitochondrial dysfunction in animal models of PTSD: Relationships between behavioral models, neural regions, and cellular maladaptation. Front. Physiol..

[12] García-Bueno B., Caso J.R., Leza J.C. (2008). Stress as a neuroinflammatory condition in brain: Damaging and protective mechanisms. Neurosci. Biobehav. Rev..

[13] Zhao W., Spiers J.G., Vassileff N., Khadka A., Jaehne E.J., van den Buuse M., Hill A.F. (2023). microRNA-146a modulates behavioural activity, neuroinflammation, and oxidative stress in adult mice. Mol. Cell. Neurosci..

[14] Schumpert C.A., Anderson C., Dudycha J.L., Patel R.C. (2016). Involvement of Daphnia pulicaria Sir2 in regulating stress response and lifespan. Aging.

[15] Liu S., Sun J., Li Y. (2015). The neuroprotective effects of resveratrol preconditioning in transient global cerebral ischemia-reperfusion in mice. Turk. Neurosurg..

[16] Porro C., Cianciulli A., Calvello R., Panaro M. (2015). Reviewing the Role of Resveratrol as a Natural Modulator of Microglial Activities. Curr. Pharm. Des..

[17] Alves-Fernandes D.K., Jasiulionis M.G. (2019). The Role of SIRT1 on DNA Damage Response and Epigenetic Alterations in Cancer. Int. J. Mol. Sci..

[18] Magnifico S., Saias L., Deleglise B., Duplus E., Kilinc D., Miquel M., Viovy J., Brugg B., Peyrin J. (2013). NAD^+^ acts on mitochondrial SirT3 to prevent axonal caspase activation and axonal degeneration. FASEB J..

[19] Renaud J., Bournival J., Zottig X., Martinoli M.G. (2013). Resveratrol Protects DAergic PC12 Cells from High Glucose-Induced Oxidative Stress and Apoptosis: Effect on p53 and GRP75 Localization. Neurotox. Res..

[20] Bureau G., Longpré F., Martinoli M. (2007). Resveratrol and quercetin, two natural polyphenols, reduce apoptotic neuronal cell death induced by neuroinflammation. J. Neurosci. Res..

[21] Sun Q., Kang R., Chen K., Liu K., Ma Z., Liu C., Deng Y., Liu W., Xu B. (2020). Sirtuin 3 is required for the protective effect of Resveratrol on Manganese-induced disruption of mitochondrial biogenesis in primary cultured neurons. J. Neurochem..

[22] Rege S., Geetha T., Broderick T., Babu J. (2015). Resveratrol Protects *β* Amyloid-Induced Oxidative Damage and Memory Associated Proteins in H19-7 Hippocampal Neuronal Cells. Curr. Alzheimer Res..

[23] Yang J., Huang J., Shen C., Cheng W., Yu P., Wang L., Tang F., Guo S., Yang Q., Zhang J. (2018). Resveratrol Treatment in Different Time-Attenuated Neuronal Apoptosis after Oxygen and Glucose Deprivation/Reoxygenation via Enhancing the Activation of Nrf-2 Signaling Pathway In Vitro. Cell Transplant..

[24] Sun D., Yue Q., Guo W., Li T., Zhang J., Li G., Liu Z., Sun J. (2015). Neuroprotection of resveratrol against neurotoxicity induced by methamphetamine in mouse mesencephalic dopaminergic neurons. BioFactors.

[25] Moldzio R., Radad K., Krewenka C., Kranner B., Duvigneau J.C., Rausch W.D. (2013). Protective effects of resveratrol on glutamate-induced damages in murine brain cultures. J. Neural Transm..

[26] Alvira D., Yeste-Velasco M., Folch J., Verdaguer E., Canudas A., Pallàs M., Camins A. (2007). Comparative analysis of the effects of resveratrol in two apoptotic models: Inhibition of complex I and potassium deprivation in cerebellar neurons. Neuroscience.

[27] Lin C.H., Nicol C.J., Cheng Y.C., Yen C., Wang Y.S., Chiang M.C. (2020). Neuroprotective effects of resveratrol against oxygen glucose deprivation induced mitochondrial dysfunction by activation of AMPK in SH-SY5Y cells with 3D gelatin scaffold. Brain Res..

[28] Rahman S., Islam R. (2011). Mammalian Sirt1: Insights on its biological functions. Cell Commun. Signal..

[29] Gertz M., Nguyen G.T.T., Fischer F., Suenkel B., Schlicker C., Fränzel B., Tomaschewski J., Aladini F., Becker C., Wolters D. (2012). A Molecular Mechanism for Direct Sirtuin Activation by Resveratrol. PLoS ONE.

[30] Cao D., Wang M., Qiu X., Liu D., Jiang H., Yang N., Xu R.M. (2015). Structural basis for allosteric, substrate-dependent stimulation of SIRT1 activity by resveratrol. Genes Dev..

[31] Chen M., Tan J., Jin Z., Jiang T., Wu J., Yu X. (2024). Research progress on Sirtuins (SIRTs) family modulators. Biomed. Pharmacother..

[32] Jęśko H., Wencel P., Strosznajder R.P., Strosznajder J.B. (2016). Sirtuins and Their Roles in Brain Aging and Neurodegenerative Disorders. Neurochem. Res..

[33] Kratz E.M., Sołkiewicz K., Kubis-Kubiak A., Piwowar A. (2021). Sirtuins as Important Factors in Pathological States and the Role of Their Molecular Activity Modulators. Int. J. Mol. Sci..

[34] Hou X., Rooklin D., Fang H., Zhang Y. (2016). Resveratrol serves as a protein-substrate interaction stabilizer in human SIRT1 activation. Sci. Rep..

[35] Ghosh S., Liu B., Zhou Z. (2013). Resveratrol activates SIRT1 in a Lamin A-dependent manner. Cell Cycle.

[36] Cantó C., Auwerx J. (2009). PGC-1*α*, SIRT1 and AMPK, an energy sensing network that controls energy expenditure. Curr. Opin. Lipidol..

[37] Zeqiraj E., Filippi B.M., Deak M., Alessi D.R., van Aalten D.M.F. (2009). Structure of the LKB1-STRAD-MO25 Complex Reveals an Allosteric Mechanism of Kinase Activation. Science.

[38] Amat R., Planavila A., Chen S.L., Iglesias R., Giralt M., Villarroya F. (2009). SIRT1 Controls the Transcription of the Peroxisome Proliferator-activated Receptor-*γ* Co-activator-1*α* (PGC-1*α*) Gene in Skeletal Muscle through the PGC-1*α* Autoregulatory Loop and Interaction with MyoD. J. Biol. Chem..

[39] Rius-Perez S., Torres-Cuevas I., Millan I., Ortega A.L., Perez S. (2020). PGC-1*α*, Inflammation, and Oxidative Stress: An Integrative View in Metabolism. Oxid. Med. Cell. Longev..

[40] Stein S.C., Woods A., Jones N.A., Davison M.D., Carling D. (2000). The regulation of AMP-activated protein kinase by phosphorylation. Biochem. J..

[41] Koronowski K.B., Khoury N., Saul I., Loris Z.B., Cohan C.H., Stradecki-Cohan H.M., Dave K.R., Young J.I., Perez-Pinzon M.A. (2017). Neuronal SIRT1 (Silent Information Regulator 2 Homologue 1) Regulates Glycolysis and Mediates Resveratrol-Induced Ischemic Tolerance. Stroke.

[42] Price N., Gomes A., Ling A., Duarte F., Martin-Montalvo A., North B., Agarwal B., Ye L., Ramadori G., Teodoro J. (2012). SIRT1 Is Required for AMPK Activation and the Beneficial Effects of Resveratrol on Mitochondrial Function. Cell Metab..

[43] Ungurianu A., Zanfirescu A., Margină D. (2023). Sirtuins, resveratrol and the intertwining cellular pathways connecting them. Ageing Res. Rev..

[44] Rasouri S., Lagouge M., Auwerx J. (2007). SIRT1/PGC-1: Un axe neuroprotecteur?. Méd. Sci..

[45] Zhang Z., Fang J., Zhou J., Ding F., Zhou G., Zhao X., Zhuang Z., Lu Y. (2022). Pterostilbene Attenuates Subarachnoid Hemorrhage-Induced Brain Injury through the SIRT1-Dependent Nrf2 Signaling Pathway. Oxid. Med. Cell. Longev..

[46] Shayganfard M. (2020). Molecular and biological functions of resveratrol in psychiatric disorders: A review of recent evidence. Cell Biosci..

[47] Kumar A., Singh C.K., LaVoie H.A., DiPette D.J., Singh U.S. (2011). Resveratrol Restores Nrf2 Level and Prevents Ethanol-Induced Toxic Effects in the Cerebellum of a Rodent Model of Fetal Alcohol Spectrum Disorders. Mol. Pharmacol..

[48] Lu X., Xu H., Sun B., Zhu Z., Zheng D., Li X. (2013). Enhanced Neuroprotective Effects of Resveratrol Delivered by Nanoparticles on Hydrogen Peroxide-Induced Oxidative Stress in Rat Cortical Cell Culture. Mol. Pharm..

[49] Pereira T.C.B., Rico E.P., Rosemberg D.B., Schirmer H., Dias R.D., Souto A.A., Bonan C.D., Bogo M.R. (2011). Zebrafish as a Model Organism to Evaluate Drugs Potentially Able to Modulate Sirtuin Expression. Zebrafish.

[50] Peñalver P., Belmonte-Reche E., Adán N., Caro M., Mateos-Martín M.L., Delgado M., González-Rey E., Morales J.C. (2018). Alkylated resveratrol prodrugs and metabolites as potential therapeutics for neurodegenerative diseases. Eur. J. Med. Chem..

[51] Kumar P., Padi S.S., Naidu P.S., Kumar A. (2006). Effect of resveratrol on 3-nitropropionic acid-induced biochemical and behavioural changes: Possible neuroprotective mechanisms. Behav. Pharmacol..

[52] Sun J., Pu C., Yang E., Zhang H., Feng Y., Luo P., Yang Y., Zhang L., Li X., Jiang X. (2023). Macrophage/Microglia Sirt3 Contributes to the Anti-inflammatory Effects of Resveratrol Against Experimental Intracerebral Hemorrhage in Mice. Cell. Mol. Neurobiol..

[53] Lee J.G., Yon J.M., Lin C., Jung A.Y., Jung K.Y., Nam S.Y. (2012). Combined treatment with capsaicin and resveratrol enhances neuroprotection against glutamate-induced toxicity in mouse cerebral cortical neurons. Food Chem. Toxicol..

[54] Khan R.S., Fonseca-Kelly Z., Callinan C., Zuo L., Sachdeva M.M., Shindler K.S. (2012). SIRT1 activating compounds reduce oxidative stress and prevent cell death in neuronal cells. Front. Cell. Neurosci..

[55] William Raja T.R., Duraipandiyan V., Ignacimuthu S., Janakiraman U., Packiam S.M. (2023). Role of Polyphenols in Alleviating Alzheimer’s Disease: A Review. Curr. Med. Chem..

[56] Wang H., Jiang T., Li W., Gao N., Zhang T. (2018). Resveratrol attenuates oxidative damage through activating mitophagy in an in vitro model of Alzheimer’s disease. Toxicol. Lett..

[57] Lagouge M., Argmann C., Gerhart-Hines Z., Meziane H., Lerin C., Daussin F., Messadeq N., Milne J., Lambert P., Elliott P. (2006). Resveratrol Improves Mitochondrial Function and Protects against Metabolic Disease by Activating SIRT1 and PGC-1*α*. Cell.

[58] Ungvari Z., Bagi Z., Feher A., Recchia F.A., Sonntag W.E., Pearson K., de Cabo R., Csiszar A. (2010). Resveratrol confers endothelial protection via activation of the antioxidant transcription factor Nrf2. Am. J.-Physiol.-Heart Circ. Physiol..

[59] Pshenichnyuk S.A., Komolov A.S. (2015). Dissociative Electron Attachment to Resveratrol as a Likely Pathway for Generation of the H2 Antioxidant Species Inside Mitochondria. J. Phys. Chem. Lett..

[60] Pollicino F., Veronese N., Dominguez L.J., Barbagallo M. (2023). Mediterranean diet and mitochondria: New findings. Exp. Gerontol..

[61] Shi L., Zhang J., Wang Y., Hao Q., Chen H., Cheng X. (2020). Sirt1 Regulates Oxidative Stress in Oxygen-Glucose Deprived Hippocampal Neurons. Front. Pediatr..

[62] Xiong H., Chen S., Lai L., Yang H., Xu Y., Pang J., Su Z., Lin H., Zheng Y. (2019). Modulation of miR-34a/SIRT1 signaling protects cochlear hair cells against oxidative stress and delays age-related hearing loss through coordinated regulation of mitophagy and mitochondrial biogenesis. Neurobiol. Aging.

[63] Wang N., Luo Z., Jin M., Sheng W., Wang H.T., Long X., Wu Y., Hu P., Xu H., Zhang X. (2019). Exploration of age-related mitochondrial dysfunction and the anti-aging effects of resveratrol in zebrafish retina. Aging.

[64] Lan F., Cacicedo J.M., Ruderman N., Ido Y. (2008). SIRT1 Modulation of the Acetylation Status, Cytosolic Localization, and Activity of LKB1. J. Biol. Chem..

[65] Cantó C., Gerhart-Hines Z., Feige J.N., Lagouge M., Noriega L., Milne J.C., Elliott P.J., Puigserver P., Auwerx J. (2009). AMPK regulates energy expenditure by modulating NAD+ metabolism and SIRT1 activity. Nature.

[66] Herzig S., Shaw R.J. (2017). AMPK: Guardian of metabolism and mitochondrial homeostasis. Nat. Rev. Mol. Cell Biol..

[67] Chen M., Yan R., Luo J., Ning J., Zhou R., Ding L. (2023). The Role of PGC-1*α*-Mediated Mitochondrial Biogenesis in Neurons. Neurochem. Res..

[68] Wu Y., Li X., Zhu J.X., Xie W., Le W., Fan Z., Jankovic J., Pan T. (2011). Resveratrol-Activated AMPK/SIRT1/Autophagy in Cellular Models of Parkinson’s Disease. Neurosignals.

[69] Feng Y., Liu T., Dong S., Guo Y., Jankovic J., Xu H., Wu Y. (2015). Rotenone affects p53 transcriptional activity and apoptosis via targeting SIRT1 and H3K9 acetylation in SH-SY5Y cells. J. Neurochem..

[70] Pineda-Ramírez N., Alquisiras-Burgos I., Ortiz-Plata A., Ruiz-Tachiquín M.E., Espinoza-Rojo M., Aguilera P. (2019). Resveratrol Activates Neuronal Autophagy Through AMPK in the Ischemic Brain. Mol. Neurobiol..

[71] Kulkarni S.S., Cantó C. (2015). The molecular targets of resveratrol. Biochim. Biophys. Acta BBA-Mol. Basis Dis..

[72] Kanthasamy K., Gordon R., Jin H., Anantharam V., Ali S., Kanthasamy G.A., Kanthasamy A. (2011). Neuroprotective Effect of Resveratrol Against Methamphetamine-Induced Dopaminergic Apoptotic Cell Death in a Cell Culture Model of Neurotoxicity. Curr. Neuropharmacol..

[73] Renaud J., Martinoli M.G. (2014). Resveratrol as a Protective Molecule for Neuroinflammation: A Review of Mechanisms. Curr. Pharm. Biotechnol..

[74] Block M.L., Zecca L., Hong J.S. (2007). Microglia-mediated neurotoxicity: Uncovering the molecular mechanisms. Nat. Rev. Neurosci..

[75] Chen J., Zhou Y., Mueller-Steiner S., Chen L.F., Kwon H., Yi S., Mucke L., Gan L. (2005). SIRT1 Protects against Microglia-dependent Amyloid-*β* Toxicity through Inhibiting NF-*κ*B Signaling. J. Biol. Chem..

[76] Zhang Q., Yuan L., Zhang Q., Gao Y., Liu G., Xiu M., Wei X., Wang Z., Liu D. (2015). Resveratrol attenuates hypoxia-induced neurotoxicity through inhibiting microglial activation. Int. Immunopharmacol..

[77] Liu J., Liao H., Chen Y., Zhu H., Li X., Liu J., Xiang Q., Zeng F., Yang Q. (2022). Resveratrol Inhibits Oxidative Stress and Regulates M1/M2-Type Polarization of Microglia via Mediation of the Nrf2/Shh Signaling Cascade after OGD/R Injury In Vitro. J. Pers. Med..

[78] Ye J., Liu Z., Wei J., Lu L., Huang Y., Luo L., Xie H. (2013). Protective effect of SIRT1 on toxicity of microglial-derived factors induced by LPS to PC12 cells via the p53-caspase-3-dependent apoptotic pathway. Neurosci. Lett..

[79] Tufekci K.U., Eltutan B.I., Isci K.B., Genc S. (2021). Resveratrol Inhibits NLRP3 Inflammasome-Induced Pyroptosis and miR-155 Expression in Microglia Through Sirt1/AMPK Pathway. Neurotox. Res..

[80] Tang X.L., Wang X., Fang G., Zhao Y.L., Yan J., Zhou Z., Sun R., Luo A.L., Li S.Y. (2021). Resveratrol ameliorates sevoflurane-induced cognitive impairment by activating the SIRT1/NF-*κ*B pathway in neonatal mice. J. Nutr. Biochem..

[81] Wan L., Jia R.M., Ji L.L., Qin X.M., Hu L., Hu F., Han Y., Pan Y.B., Jiang C.Y., Liu W.T. (2022). AMPK-autophagy-mediated inhibition of microRNA-30a-5p alleviates morphine tolerance via SOCS3-dependent neuroinflammation suppression. J. Neuroinflamm..

[82] Ueki K., Kondo T., Kahn C.R. (2004). Suppressor of Cytokine Signaling 1 (SOCS-1) and SOCS-3 Cause Insulin Resistance through Inhibition of Tyrosine Phosphorylation of Insulin Receptor Substrate Proteins by Discrete Mechanisms. Mol. Cell. Biol..

[83] Han X., Chen X., Han J., Zhong Y., Li Q., An Y. (2020). MiR-324/SOCS3 Axis Protects Against Hypoxia/Reoxygenation-Induced Cardiomyocyte Injury and Regulates Myocardial Ischemia via TNF/NF-*κ*B Signaling Pathway. Int. Heart J..

[84] Ravanan P., Srikumar I.F., Talwar P. (2017). Autophagy: The spotlight for cellular stress responses. Life Sci..

[85] Ng F. (2015). SIRT1 in the brain—Connections with aging-associated disorders and lifespan. Front. Cell. Neurosci..

[86] Cavaliere G., Trinchese G., Penna E., Cimmino F., Pirozzi C., Lama A., Annunziata C., Catapano A., Mattace Raso G., Meli R. (2019). High-Fat Diet Induces Neuroinflammation and Mitochondrial Impairment in Mice Cerebral Cortex and Synaptic Fraction. Front. Cell. Neurosci..

[87] Kauer-Sant’Anna M., Kapczinski F., Andreazza A.C., Bond D.J., Lam R.W., Young L.T., Yatham L.N. (2008). Brain-derived neurotrophic factor and inflammatory markers in patients with early- vs. late-stage bipolar disorder. Int. J. Neuropsychopharmacol..

[88] Ding H., Chen J., Su M., Lin Z., Zhan H., Yang F., Li W., Xie J., Huang Y., Liu X. (2020). BDNF promotes activation of astrocytes and microglia contributing to neuroinflammation and mechanical allodynia in cyclophosphamide-induced cystitis. J. Neuroinflamm..

[89] Zhang F., Lu Y.F., Wu Q., Liu J., Shi J.S. (2012). Resveratrol promotes neurotrophic factor release from astroglia. Exp. Biol. Med..

[90] Shen J., Xu L., Qu C., Sun H., Zhang J. (2018). Resveratrol prevents cognitive deficits induced by chronic unpredictable mild stress: Sirt1/miR-134 signalling pathway regulates CREB/BDNF expression in hippocampus in vivo and in vitro. Behav. Brain Res..

[91] Zhang F., Wang Y.Y., Liu H., Lu Y.F., Wu Q., Liu J., Shi J.S. (2012). Resveratrol Produces Neurotrophic Effects on Cultured Dopaminergic Neurons through Prompting Astroglial BDNF and GDNF Release. Evid.-Based Complement. Altern. Med..

[92] Hsieh C.P., Chang W.T., Chen L., Chen H.H., Chan M.H. (2019). Differential inhibitory effects of resveratrol on excitotoxicity and synaptic plasticity: Involvement of NMDA receptor subtypes. Nutr. Neurosci..

[93] Cui S.Y., Yang M.X., Zhang Y.H., Zheng V., Zhang H.T., Gurney M.E., Xu Y., O’Donnell J.M. (2019). Protection from Amyloid *β* Peptide–Induced Memory, Biochemical, and Morphological Deficits by a Phosphodiesterase-4D Allosteric Inhibitor. J. Pharmacol. Exp. Ther..

[94] Lissin D.V., Carroll R.C., Nicoll R.A., Malenka R.C., Zastrow M.V. (1999). Rapid, Activation-Induced Redistribution of Ionotropic Glutamate Receptors in Cultured Hippocampal Neurons. J. Neurosci..

[95] Kim J.H., Chung K.H., Hwang Y.R., Park H.R., Kim H.J., Kim H.G., Kim H.R. (2021). Exposure to RF-EMF Alters Postsynaptic Structure and Hinders Neurite Outgrowth in Developing Hippocampal Neurons of Early Postnatal Mice. Int. J. Mol. Sci..

[96] Liu X., Tang M., He T.Y., Zhao S., Li H.Z., Li Z., Guo Y.X., Wang X.L. (2023). Resveratrol Improves Paclitaxel-Induced Cognitive Impairment in Mice by Activating SIRT1/PGC-1*α* Pathway to Regulate Neuronal State and Microglia Cell Polarization. Drug Des. Dev. Ther..

[97] Wei Y.D., Chen X.X., Yang L.J., Gao X.R., Xia Q.R., Qi C.C., Ge J.F. (2022). Resveratrol ameliorates learning and memory impairments induced by bilateral hippocampal injection of streptozotocin in mice. Neurochem. Int..

[98] Saviola F., Pappaianni E., Monti A., Grecucci A., Jovicich J., De Pisapia N. (2020). Trait and state anxiety are mapped differently in the human brain. Sci. Rep..

[99] Govic A., Nasser H., Levay E.A., Zelko M., Ebrahimie E., Mohammadi Dehcheshmeh M., Kent S., Penman J., Hazi A. (2022). Long-Term Calorie Restriction Alters Anxiety-like Behaviour and the Brain and Adrenal Gland Transcriptomes of the Ageing Male Rat. Nutrients.

[100] Lu K., Jia X., Wu J., Wang Q., Liang X.F. (2023). Neuropeptide Y receptor Y2 (npy2r) deficiency reduces anxiety and increases food intake in Japanese medaka (*Oryzias latipes*). Front. Cell Dev. Biol..

[101] Robinette T.M., Nicholatos J.W., Francisco A.B., Brooks K.E., Diao R.Y., Sorbi S., Ricca V., Nacmias B., Brieño-Enríquez M.A., Libert S. (2020). SIRT1 accelerates the progression of activity-based anorexia. Nat. Commun..

[102] Staples L.G. (2010). Predator odor avoidance as a rodent model of anxiety: Learning-mediated consequences beyond the initial exposure. Neurobiol. Learn. Mem..

[103] Shekhar A., Truitt W., Rainnie D., Sajdyk T. (2005). Role of stress, corticotrophin releasing factor (CRF) and amygdala plasticity in chronic anxiety. Stress.

[104] Musazzi L., Tornese P., Sala N., Popoli M. (2018). What Acute Stress Protocols Can Tell Us About PTSD and Stress-Related Neuropsychiatric Disorders. Front. Pharmacol..

[105] Liu Y., Zhao J., Guo W. (2018). Emotional Roles of Mono-Aminergic Neurotransmitters in Major Depressive Disorder and Anxiety Disorders. Front. Psychol..

[106] Kraeuter A.K., Guest P.C., Sarnyai Z. (2018). The Elevated Plus Maze Test for Measuring Anxiety-Like Behavior in Rodents. Pre-Clinical Models.

[107] de Boer S.F., Buwalda B., Koolhaas J.M. (2017). Untangling the neurobiology of coping styles in rodents: Towards neural mechanisms underlying individual differences in disease susceptibility. Neurosci. Biobehav. Rev..

[108] Ullmann E., Perry S.W., Licinio J., Wong M.L., Dremencov E., Zavjalov E.L., Shevelev O.B., Khotskin N.V., Koncevaya G.V., Khotshkina A.S. (2019). From Allostatic Load to Allostatic State—An Endogenous Sympathetic Strategy to Deal with Chronic Anxiety and Stress?. Front. Behav. Neurosci..

[109] Tseilikman O.B., Kozochkin D.A., Manukhina E.B., Downey H.F., Misharina M.E., Komelkova M.V., Nikitina A.A., Golodnii S.V., Dodohova M.A., Tseilikman V.E. (2016). Predicting anxiety responses to halogenated glucocorticoid drugs using the hexobarbital sleep time test. Stress.

[110] Meier S.M., Deckert J. (2019). Genetics of Anxiety Disorders. Curr. Psychiatry Rep..

[111] Tseilikman V.E., Tseilikman O.B., Pashkov A.A., Ivleva I.S., Karpenko M.N., Shatilov V.A., Zhukov M.S., Fedotova J.O., Kondashevskaya M.V., Downey H.F. (2022). Mechanisms of Susceptibility and Resilience to PTSD: Role of Dopamine Metabolism and BDNF Expression in the Hippocampus. Int. J. Mol. Sci..

[112] Rao U. (2010). Comorbidity between depressive and addictive disorders in adolescents: Role of stress and hpa activity. US Psyc..

[113] Calhoon G.G., Tye K.M. (2015). Resolving the neural circuits of anxiety. Nat. Neurosci..

[114] Jarrin S., Finn D.P. (2019). Optogenetics and its application in pain and anxiety research. Neurosci. Biobehav. Rev..

[115] Britt J., McDevitt R., Reed S. (2014). Optogenetics in preclinical neuroscience and psychiatry research: Recent insights and potential applications. Neuropsychiatr. Dis. Treat..

[116] Alexandra Kredlow M., Fenster R.J., Laurent E.S., Ressler K.J., Phelps E.A. (2021). Prefrontal cortex, amygdala, and threat processing: Implications for PTSD. Neuropsychopharmacology.

[117] Akirav I., Maroun M. (2007). The Role of the Medial Prefrontal Cortex-Amygdala Circuit in Stress Effects on the Extinction of Fear. Neural Plast..

[118] Perumal M.B., Sah P. (2021). Inhibitory Circuits in the Basolateral Amygdala in Aversive Learning and Memory. Front. Neural Circuits.

[119] Lu Y., Simpson K.L., Weaver K.J., Lin R.C. (2012). Differential Distribution Patterns From Medial Prefrontal Cortex and Dorsal Raphe to the Locus Coeruleus in Rats. Anat. Rec..

[120] Marek R., Strobel C., Bredy T.W., Sah P. (2013). The amygdala and medial prefrontal cortex: Partners in the fear circuit. J. Physiol..

[121] McEwen B.S., Nasca C., Gray J.D. (2015). Stress Effects on Neuronal Structure: Hippocampus, Amygdala, and Prefrontal Cortex. Neuropsychopharmacology.

[122] Olivier J.D.A., Olivier B. (2020). Translational Studies in the Complex Role of Neurotransmitter Systems in Anxiety and Anxiety Disorders. Anxiety Disorders.

[123] Ardianto C., Budiatin A.S., Sumartha I.N.B., Nurrahmi N., Rahmadi M., Khotib J. (2021). Resveratrol ameliorates physical and psychological stress-induced depressive-like behavior. J. Basic Clin. Physiol. Pharmacol..

[124] Gao Z.B., Chen X.Q., Hu G.Y. (2006). Inhibition of excitatory synaptic transmission by trans-resveratrol in rat hippocampus. Brain Res..

[125] Ro J.H., Liu C.C., Lin M.C. (2020). Resveratrol Mitigates Cerebral Ischemic Injury by Altering Levels of Trace Elements, Toxic Metal, Lipid Peroxidation, and Antioxidant Activity. Biol. Trace Elem. Res..

[126] Parveen A., Alqahtani F., Javaid S., Ashraf W., Siddique F., Rawat R., Rasool M.F., Ahmad T., Alasmari F., Imran I. (2023). Anxiolytic potential of resveratrol and rufinamide combination by modulating GABA-ergic transmission: Insights from experiments, molecular docking and dynamics simulations. J. Physiol. Pharmacol..

[127] Sarubbo F., Ramis M.R., Aparicio S., Ruiz L., Esteban S., Miralles A., Moranta D. (2015). Improving effect of chronic resveratrol treatment on central monoamine synthesis and cognition in aged rats. AGE.

[128] Shuto T., Kuroiwa M., Koga Y., Kawahara Y., Sotogaku N., Toyomasu K., Nishi A. (2013). Acute effects of resveratrol to enhance cocaine-induced dopamine neurotransmission in the striatum. Neurosci. Lett..

[129] Gu Z., Chu L., Han Y. (2019). Therapeutic effect of resveratrol on mice with depression. Exp. Ther. Med..

[130] Sun Z., Liu S., Kharlamov E.A., Miller E.R., Kelly K.M. (2018). Hippocampal neuropeptide Y protein expression following controlled cortical impact and posttraumatic epilepsy. Epilepsy Behav..

[131] Nwokafor C., Serova L.I., Nahvi R.J., McCloskey J., Sabban E.L. (2020). Activation of NPY receptor subtype 1 by [D-His26]NPY is sufficient to prevent development of anxiety and depressive like effects in the single prolonged stress rodent model of PTSD. Neuropeptides.

[132] Harada N., Zhao J., Kurihara H., Nakagata N., Okajima K. (2011). Resveratrol improves cognitive function in mice by increasing production of insulin-like growth factor-I in the hippocampus. J. Nutr. Biochem..

[133] Tong J., Gao J., Liu Q., He C., Zhao X., Qi Y., Yuan T., Li P., Niu M., Wang D. (2020). Resveratrol derivative excited postsynaptic potentiation specifically via PKC*β*-NMDA receptor mediation. Pharmacol. Res..

[134] Kondashevskaya M.V., Downey H.F., Tseilikman V.E., Alexandrin V.V., Artem’yeva K.A., Aleksankina V.V., Tseilikman O.B., Pashkov A.A., Goryacheva A.V., Ivleva I.S. (2022). Cerebral Blood Flow in Predator Stress-Resilient and -Susceptible Rats and Mechanisms of Resilience. Int. J. Mol. Sci..

[135] Tseilikman V., Akulov A., Shevelev O., Khotskina A., Kontsevaya G., Moshkin M., Fedotova J., Pashkov A., Tseilikman O., Agletdinov E. (2022). Paradoxical Anxiety Level Reduction in Animal Chronic Stress: A Unique Role of Hippocampus Neurobiology. Int. J. Mol. Sci..

[136] Endo Y., Nishimura J.I., Kobayashi S., Kimura F. (1997). Long-term glucocorticoid treatments decrease local cerebral blood flow in the rat hippocampus, in association with histological damage. Neuroscience.

[137] Hattori Y., Kakino Y., Hattori Y., Iwashita M., Uchiyama H., Noda K., Yoshimoto T., Iida H., Ihara M. (2024). Long-Term Resveratrol Intake for Cognitive and Cerebral Blood Flow Impairment in Carotid Artery Stenosis/Occlusion. J. Stroke.

[138] Garrigue P., Mounien L., Champion S., Mouhajir Y., Pechere L., Guillet B., Landrier J.F., Seree E. (2021). Long-term administration of resveratrol at low doses improves neurocognitive performance as well as cerebral blood flow and modulates the inflammatory pathways in the brain. J. Nutr. Biochem..

[139] Ji N., Lei M., Chen Y., Tian S., Li C., Zhang B. (2023). How Oxidative Stress Induces Depression?. ASN Neuro.

[140] Leal G., Comprido D., Duarte C.B. (2014). BDNF-induced local protein synthesis and synaptic plasticity. Neuropharmacology.

[141] Lee C.W., Fang Y.P., Chu M.C., Chung Y.J., Chi H., Tang C.W., So E.C., Lin H.C., Lin H.C. (2021). Differential mechanisms of synaptic plasticity for susceptibility and resilience to chronic social defeat stress in male mice. Biochem. Biophys. Res. Commun..

[142] Dong E., Guidotti A., Zhang H., Pandey S.C. (2018). Prenatal stress leads to chromatin and synaptic remodeling and excessive alcohol intake comorbid with anxiety-like behaviors in adult offspring. Neuropharmacology.

[143] Yu Y., Xu D., Cheng S., Zhang L., Shi Z., Qin J., Zhang Z., Wang H. (2019). Prenatal ethanol exposure enhances the susceptibility to depressive behavior of adult offspring rats fed a high-fat diet by affecting BDNF-associated pathway. Int. J. Mol. Med..

[144] Fan B., Hao B., Dai Y., Xue L., Shi Y., Liu L., Xuan S., Yang N., Wang X., Zhao H. (2022). Deficiency of Tet3 in nucleus accumbens enhances fear generalization and anxiety-like behaviors in mice. Brain Pathol..

[145] Yang X.H., Song S.Q., Xu Y. (2017). Resveratrol ameliorates chronic unpredictable mild stress-induced depression-like behavior: Involvement of the HPA axis, inflammatory markers, BDNF, and Wnt/*β*-catenin pathway in rats. Neuropsychiatr. Dis. Treat..

[146] Liaqat H., Parveen A., Kim S.Y. (2022). Antidepressive Effect of Natural Products and Their Derivatives Targeting BDNF-TrkB in Gut–Brain Axis. Int. J. Mol. Sci..

[147] Mehta K., Pandey K.K., Kaur B., Dhar P., Kaler S. (2021). Resveratrol attenuates arsenic-induced cognitive deficits via modulation of Estrogen-NMDAR-BDNF signalling pathway in female mouse hippocampus. Psychopharmacology.

[148] Lv X., Chen S., Gao F., Hu B., Wang Y., Ni S., Kou H., Song Z., Qing X., Wang S. (2021). Resveratrol-enhanced SIRT1-mediated osteogenesis in porous endplates attenuates low back pain and anxiety behaviors. FASEB J..

[149] Xu Y., Cui S.Y., Ma Q., Shi J., Yu Y., Li J.X., Zheng L., Zhang Y., Si J.M., Yu Y.C. (2018). trans-Resveratrol Ameliorates Stress-Induced Irritable Bowel Syndrome-Like Behaviors by Regulation of Brain-Gut Axis. Front. Pharmacol..

[150] Abe-Higuchi N., Uchida S., Yamagata H., Higuchi F., Hobara T., Hara K., Kobayashi A., Watanabe Y. (2016). Hippocampal Sirtuin 1 Signaling Mediates Depression-like Behavior. Biol. Psychiatry.

[151] Zhu X., Li W., Li Y., Xu W., Yuan Y., Zheng V., Zhang H., O’Donnell J.M., Xu Y., Yin X. (2019). The antidepressant- and anxiolytic-like effects of resveratrol: Involvement of phosphodiesterase-4D inhibition. Neuropharmacology.

[152] Barnes P.J. (2010). Mechanisms and resistance in glucocorticoid control of inflammation. J. Steroid Biochem. Mol. Biol..

[153] Mourtzi N., Sertedaki A., Charmandari E. (2021). Glucocorticoid Signaling and Epigenetic Alterations in Stress-Related Disorders. Int. J. Mol. Sci..

[154] Sarapultsev A., Sarapultsev P., Dremencov E., Komelkova M., Tseilikman O., Tseilikman V. (2020). Low glucocorticoids in stress-related disorders: The role of inflammation. Stress.

[155] Maloley P., England B., Sayles H., Thiele G., Michaud K., Sokolove J., Cannon G., Reimold A., Kerr G., Baker J. (2019). Post-traumatic stress disorder and serum cytokine and chemokine concentrations in patients with rheumatoid arthritis. Semin. Arthritis Rheum..

[156] Lindqvist D., Dhabhar F.S., Mellon S.H., Yehuda R., Grenon S.M., Flory J.D., Bierer L.M., Abu-Amara D., Coy M., Makotkine I. (2017). Increased pro-inflammatory milieu in combat related PTSD—A new cohort replication study. Brain Behav. Immun..

[157] Fairless R., Bading H., Diem R. (2021). Pathophysiological Ionotropic Glutamate Signalling in Neuroinflammatory Disease as a Therapeutic Target. Front. Neurosci..

[158] Zhu C.B., Blakely R.D., Hewlett W.A. (2006). The Proinflammatory Cytokines Interleukin-1beta and Tumor Necrosis Factor-Alpha Activate Serotonin Transporters. Neuropsychopharmacology.

[159] Shen J.D., Zhang Y.W., Wang B.Y., Bai L., Lu S.F., Zhu L.L., Bai M., Li Y.C., Xu E.P. (2020). Effects of resveratrol on the levels of ATP, 5-HT and GAP-43 in the hippocampus of mice exposed to chronic unpredictable mild stress. Neurosci. Lett..

[160] Zarebavani M., Baghaei Naeini F., Farahvash A., Moradi F., Dashti N. (2023). Resveratrol attenuates chronic social isolation stress-induced affective disorders: Involvement of NF-*κ*B/NLRP3 axis. J. Biochem. Mol. Toxicol..

[161] Wei R.M., Zhang Y.M., Feng Y.Z., Zhang K.X., Zhang J.Y., Chen J., Luo B.L., Li X.Y., Chen G.H. (2023). Resveratrol ameliorates maternal separation-induced anxiety- and depression-like behaviors and reduces Sirt1-NF-kB signaling-mediated neuroinflammation. Front. Behav. Neurosci..

[162] Molla B., Heredia M., Campos A., Sanz P. (2022). Pharmacological Modulation of Glutamatergic and Neuroinflammatory Pathways in a Lafora Disease Mouse Model. Mol. Neurobiol..

[163] Tian Q., Fan X., Ma J., Han Y., Li D., Jiang S., Zhang F., Guang H., Shan X., Chen R. (2020). Resveratrol ameliorates lipopolysaccharide-induced anxiety-like behavior by attenuating YAP-mediated neuro-inflammation and promoting hippocampal autophagy in mice. Toxicol. Appl. Pharmacol..

[164] Ploski J.E., Vaidya V.A. (2021). The Neurocircuitry of Posttraumatic Stress Disorder and Major Depression: Insights Into Overlapping and Distinct Circuit Dysfunction—A Tribute to Ron Duman. Biol. Psychiatry.

[165] Sharma A., Liaw K., Sharma R., Zhang Z., Kannan S., Kannan R.M. (2018). Targeting Mitochondrial Dysfunction and Oxidative Stress in Activated Microglia using Dendrimer-Based Therapeutics. Theranostics.

[166] Javani G., Babri S., Farajdokht F., Ghaffari-Nasab A., Mohaddes G. (2022). Mitochondrial transplantation improves anxiety- and depression-like behaviors in aged stress-exposed rats. Mech. Ageing Dev..

[167] Larrieu T., Cherix A., Duque A., Rodrigues J., Lei H., Gruetter R., Sandi C. (2017). Hierarchical Status Predicts Behavioral Vulnerability and Nucleus Accumbens Metabolic Profile Following Chronic Social Defeat Stress. Curr. Biol..

[168] Hollis F., van der Kooij M.A., Zanoletti O., Lozano L., Cantó C., Sandi C. (2015). Mitochondrial function in the brain links anxiety with social subordination. Proc. Natl. Acad. Sci. USA.

[169] Papilloud A., Weger M., Bacq A., Zalachoras I., Hollis F., Larrieu T., Battivelli D., Grosse J., Zanoletti O., Parnaudeau S. (2020). The glucocorticoid receptor in the nucleus accumbens plays a crucial role in social rank attainment in rodents. Psychoneuroendocrinology.

[170] Filiou M.D., Sandi C. (2019). Anxiety and Brain Mitochondria: A Bidirectional Crosstalk. Trends Neurosci..

[171] Picard M., McEwen B.S., Epel E.S., Sandi C. (2018). An energetic view of stress: Focus on mitochondria. Front. Neuroendocrinol..

[172] Picard M., McEwen B.S. (2013). Mitochondria impact brain function and cognition. Proc. Natl. Acad. Sci. USA.

[173] Zhao J., Ye L., Liu Z., Cui Y., Deng D., Bai S., Yang L., Shi Y., Liu Z., Zhang R. (2022). Protective Effects of Resveratrol on Adolescent Social Isolation-Induced Anxiety-Like Behaviors via Modulating Nucleus Accumbens Spine Plasticity and Mitochondrial Function in Female Rats. Nutrients.

[174] Kim H.D., Hesterman J., Call T., Magazu S., Keeley E., Armenta K., Kronman H., Neve R.L., Nestler E.J., Ferguson D. (2016). SIRT1 Mediates Depression-Like Behaviors in the Nucleus Accumbens. J. Neurosci..

[175] Tabassum S., Misrani A., Huang H.X., Zhang Z.Y., Li Q.W., Long C. (2023). Resveratrol Attenuates Chronic Unpredictable Mild Stress-Induced Alterations in the SIRT1/PGC1*α*/SIRT3 Pathway and Associated Mitochondrial Dysfunction in Mice. Mol. Neurobiol..

[176] Hovatta I., Juhila J., Donner J. (2010). Oxidative stress in anxiety and comorbid disorders. Neurosci. Res..

[177] Lim H.S., Lee S.H., Seo H., Park G. (2023). Changes in RBM47 expression based on the timing of melatonin administration and its effects on Nrf2 activity in the hippocampus. Free. Radic. Biol. Med..

[178] Khalifeh S., Oryan S., Digaleh H., Shaerzadeh F., Khodagholi F., Maghsoudi N., Zarrindast M.R. (2014). Involvement of Nrf2 in Development of Anxiety-Like Behavior by Linking Bcl2 to Oxidative Phosphorylation: Estimation in Rat Hippocampus, Amygdala, and Prefrontal Cortex. J. Mol. Neurosci..

[179] Schriever S.C., Zimprich A., Pfuhlmann K., Baumann P., Giesert F., Klaus V., Kabra D.G., Hafen U., Romanov A., Tschöp M.H. (2017). Alterations in neuronal control of body weight and anxiety behavior by glutathione peroxidase 4 deficiency. Neuroscience.

[180] Olsen R.H.J., Johnson L.A., Zuloaga D.G., Limoli C.L., Raber J. (2013). Enhanced hippocampus-dependent memory and reduced anxiety in mice over-expressing human catalase in mitochondria. J. Neurochem..

[181] Khalifeh S., Oryan S., Khodagholi F., Digaleh H., Shaerzadeh F., Maghsoudi N., Zarrindast M.R. (2015). Complexity of Compensatory Effects in Nrf1 Knockdown: Linking Undeveloped Anxiety-Like Behavior to Prevented Mitochondrial Dysfunction and Oxidative Stress. Cell. Mol. Neurobiol..

[182] Sergeeva S., Bagryanskaya E., Korbolina E., Kolosova N. (2006). Development of behavioural dysfunctions in accelerated-senescence OXYS rats is associated with early postnatal alterations in brain phosphate metabolism. Exp. Gerontol..

[183] Sharma R., Kumarasamy M., Parihar V.K., Ravichandiran V., Kumar N. (2024). Monoamine Oxidase: A Potential Link in Papez Circuit to Generalized Anxiety Disorders. Cns Neurol. Disord.-Drug Targets.

[184] Geng X., Wu H., Li Z., Li C., Chen D., Zong J., Liu Z., Wei S., Peng W. (2021). Jie-Yu-He-Huan Capsule Ameliorates Anxiety-Like Behaviours in Rats Exposed to Chronic Restraint Stress via the cAMP/PKA/CREB/BDNF Signalling Pathway. Oxid. Med. Cell. Longev..

[185] Liu M., Ling Y., Zhang Y., Liu L., Qiu Y., Liu Y., Yin Y. (2023). The role of EndophilinA1 in chronic unpredicted mild stress-induced depression model mice. Int. Immunopharmacol..

[186] Oettinghaus B., Schulz J.M., Restelli L.M., Licci M., Savoia C., Schmidt A., Schmitt K., Grimm A., Morè L., Hench J. (2015). Synaptic dysfunction, memory deficits and hippocampal atrophy due to ablation of mitochondrial fission in adult forebrain neurons. Cell Death Differ..

[187] Chiu W., Chen C., Lee T., Chen Z., Ke P., Chiang A. (2010). Oxidative stress enhances AP-1 and NF-*κ*B-mediated regulation of *β*2-Glycoprotein I gene expression in hepatoma cells. J. Cell. Biochem..

[188] Abdel-Wahab B.A., Abdel-Wahab M.M. (2016). Protective effect of resveratrol against chronic intermittent hypoxia-induced spatial memory deficits, hippocampal oxidative DNA damage and increased p47Phox NADPH oxidase expression in young rats. Behav. Brain Res..

[189] Baghaei Naeini F., Hassanpour S., Asghari A. (2023). Resveratrol exerts anxiolytic-like effects through anti-inflammatory and antioxidant activities in rats exposed to chronic social isolation. Behav. Brain Res..

[190] Shukla P., Akotkar L., Aswar U. (2024). Resveratrol attenuates early life stress induced depression in rats: Behavioural and neurochemical evidence. Neurosci. Lett..

[191] López J.F., Akil H., Watson S.J. (1999). Neural circuits mediating stress. Biol. Psychiatry.

[192] de Kloet E.R., de Kloet S.F., de Kloet C.S., de Kloet A.D. (2019). Top-down and bottom-up control of stress-coping. J. Neuroendocrinol..

[193] de Kloet E.R., Derijk R. (2004). Signaling Pathways in Brain Involved in Predisposition and Pathogenesis of Stress-Related Disease: Genetic and Kinetic Factors Affecting the MR/GR Balance. Ann. N. Y. Acad. Sci..

[194] Adcock I.M., Cosio B., Tsaprouni L., Barnes P.J., Ito K. (2005). Redox Regulation of Histone Deacetylases and Glucocorticoid-Mediated Inhibition of the Inflammatory Response. Antioxid. Redox Signal..

[195] Sánchez-Lafuente C.L., Kalynchuk L.E., Caruncho H.J., Ausió J. (2022). The Role of MeCP2 in Regulating Synaptic Plasticity in the Context of Stress and Depression. Cells.

[196] Popoli M., Yan Z., McEwen B.S., Sanacora G. (2011). The stressed synapse: The impact of stress and glucocorticoids on glutamate transmission. Nat. Rev. Neurosci..

[197] Numakawa T., Kajihara R. (2024). An Interaction between Brain-Derived Neurotrophic Factor and Stress-Related Glucocorticoids in the Pathophysiology of Alzheimer’s Disease. Int. J. Mol. Sci..

[198] Lambert W.M., Xu C.F., Neubert T.A., Chao M.V., Garabedian M.J., Jeanneteau F.D. (2013). Brain-Derived Neurotrophic Factor Signaling Rewrites the Glucocorticoid Transcriptome via Glucocorticoid Receptor Phosphorylation. Mol. Cell. Biol..

[199] Kelly J.R., Kennedy P.J., Cryan J.F., Dinan T.G., Clarke G., Hyland N.P. (2015). Breaking down the barriers: The gut microbiome, intestinal permeability and stress-related psychiatric disorders. Front. Cell. Neurosci..

[200] Yehuda R., Seckl J. (2011). Minireview: Stress-Related Psychiatric Disorders with Low Cortisol Levels: A Metabolic Hypothesis. Endocrinology.

[201] Tseilikman V., Dremencov E., Maslennikova E., Ishmatova A., Manukhina E., Downey H.F., Klebanov I., Tseilikman O., Komelkova M., Lapshin M.S. (2019). Post-Traumatic Stress Disorder Chronification via Monoaminooxidase and Cortisol Metabolism. Horm. Metab. Res..

[202] Zhang Z.S., Qiu Z.K., He J.L., Liu X., Chen J.S., Wang Y.L. (2017). Resveratrol ameliorated the behavioral deficits in a mouse model of post-traumatic stress disorder. Pharmacol. Biochem. Behav..

[203] Carabotti M., Scirocco A., Maselli M.A., Severi C. (2015). The gut-brain axis: Interactions between enteric microbiota, central and enteric nervous systems. Ann. Gastroenterol..

[204] Kuwahara A., Matsuda K., Kuwahara Y., Asano S., Inui T., Marunaka Y. (2020). Microbiota-gut-brain axis: Enteroendocrine cells and the enteric nervous system form an interface between the microbiota and the central nervous system. Biomed. Res..

[205] Zhang H., Wang Z., Wang G., Song X., Qian Y., Liao Z., Sui L., Ai L., Xia Y. (2023). Understanding the Connection between Gut Homeostasis and Psychological Stress. J. Nutr..

[206] Petakh P., Oksenych V., Kamyshna I., Boisak I., Lyubomirskaya K., Kamyshnyi O. (2024). Exploring the interplay between posttraumatic stress disorder, gut microbiota, and inflammatory biomarkers: A comprehensive meta-analysis. Front. Immunol..

[207] Yadav S.K., Ahmad R., Moshfegh C.M., Sankarasubramanian J., Joshi V., Elkhatib S.K., Chhonker Y.S., Murry D.J., Talmon G.A., Guda C. (2023). Repeated Social Defeat Stress Induces an Inflammatory Gut Milieu by Altering the Mucosal Barrier Integrity and Gut Microbiota Homeostasis. Biol. Psychiatry Glob. Open Sci..

[208] Jing W., Bi C., Fang Z., Qian C., Chen J., Yu J., Tian G., Ye M., Liu Z. (2023). Neuropsychiatric sequelae after liver transplantation and their possible mechanism via the microbiota–gut–liver–brain axis. Biomed. Pharmacother..

[209] Russo S., Chan K., Li L., Parise L., Cathomas F., LeClair K., Shimo Y., Lin H.Y., Durand-de Cuttoli R., Aubry A. (2023). Stress-activated brain-gut circuits disrupt intestinal barrier integrity and social behaviour. Res. Sq..

[210] Li L.F., Zou H.W., Song B.L., Wang Y., Jiang Y., Li Z.L., Niu Q.H., Liu Y.J. (2022). Increased Lactobacillus Abundance Contributes to Stress Resilience in Mice Exposed to Chronic Social Defeat Stress. Neuroendocrinology.

[211] Liu X., Li X., Teng T., Jiang Y., Xiang Y., Fan L., Yu Y., Zhou X., Xie P. (2022). Comparative analysis of gut microbiota and fecal metabolome features among multiple depressive animal models. J. Affect. Disord..

[212] Yu Y.C., Li J., Zhang M., Pan J.C., Yu Y., Zhang J.B., Zheng L., Si J.M., Xu Y. (2019). Resveratrol Improves Brain-Gut Axis by Regulation of 5-HT-Dependent Signaling in the Rat Model of Irritable Bowel Syndrome. Front. Cell. Neurosci..

[213] Favari C., Rinaldi de Alvarenga J.F., Sánchez-Martínez L., Tosi N., Mignogna C., Cremonini E., Manach C., Bresciani L., Del Rio D., Mena P. (2024). Factors driving the inter-individual variability in the metabolism and bioavailability of (poly)phenolic metabolites: A systematic review of human studies. Redox Biol..

[214] Tseilikman V.E., Fedotova J.O., Tseilikman O.B., Novak J., Karpenko M.N., Maistrenko V.A., Lazuko S.S., Belyeva L.E., Kamel M., Buhler A.V. (2023). Resistance to Resveratrol Treatment in Experimental PTSD Is Associated with Abnormalities in Hepatic Metabolism of Glucocorticoids. Int. J. Mol. Sci..

[215] Chen Y., Li J., Zhang M., Yang W., Qin W., Zheng Q., Chu Y., Wu Y., Wu D., Yuan X. (2022). 11*β*-HSD1 Inhibitor Alleviates Non-Alcoholic Fatty Liver Disease by Activating the AMPK/SIRT1 Signaling Pathway. Nutrients.

[216] Novak J., Tseilikman V.E., Tseilikman O.B., Lazuko S.S., Belyeva L.E., Rahmani A., Fedotova J. (2023). Can Resveratrol Influence the Activity of 11*β*-Hydroxysteroid Dehydrogenase Type 1? A Combined In Silico and In Vivo Study. Pharmaceuticals.

[217] Chistiakov D.A., Bobryshev Y.V., Chekhonin V.P. (2018). Epigenetic Alterations in DNA and Histone Modifications Caused by Depression and Antidepressant Drugs: Lessons from the Rodent Models. Curr. Pharm. Des..

[218] Kovanen L., Donner K., Partonen T. (2015). SIRT1 Polymorphisms Associate with Seasonal Weight Variation, Depressive Disorders, and Diastolic Blood Pressure in the General Population. PLoS ONE.

[219] Xu L., Xu S., Lin L., Gu X., Fu C., Fang Y., Li X., Wang X. (2018). High-fat Diet Mediates Anxiolytic-like Behaviors in a Time-dependent Manner Through the Regulation of SIRT1 in the Brain. Neuroscience.

[220] Dang R., Wang M., Li X., Wang H., Liu L., Wu Q., Zhao J., Ji P., Zhong L., Licinio J. (2022). Edaravone ameliorates depressive and anxiety-like behaviors via Sirt1/Nrf2/HO-1/Gpx4 pathway. J. Neuroinflamm..

[221] Dai G.L., Yang X.Y., Chen S.S., Wang Y.Q., Liu M.C., Cao Y., Li F.R., Ma C.Y., Ju W.Z. (2021). Effects of Jiaotai Pills on CUMS-induced depression model in mice based on changes of SIRT1 expression in hippocampus. Zhongguo Zhong Yao Zhi.

[222] Fan J., Guang H., Zhang H., Chen D., Ding L., Fan X., Xue F., Gan Z., Wang Y., Mao S. (2018). SIRT1 Mediates Apelin-13 in Ameliorating Chronic Normobaric Hypoxia-induced Anxiety-like Behavior by Suppressing NF-*κ*B Pathway in Mice Hippocampus. Neuroscience.

[223] Brynildsen J.K., Lee B.G., Perron I.J., Jin S., Kim S.F., Blendy J.A. (2018). Activation of AMPK by metformin improves withdrawal signs precipitated by nicotine withdrawal. Proc. Natl. Acad. Sci. USA.

[224] Wang J., Zhao P., Cheng P., Zhang Z., Yang S., Wang J., Wang X., Zhu G. (2024). Exploring the effect of Anshen Dingzhi prescription on hippocampal mitochondrial signals in single prolonged stress mouse model. J. Ethnopharmacol..

[225] Wang J., Ma S.F., Yun Q., Liu W.J., Guo M.N., Zhu Y.Q., Liu Z.Z., Qian J.J., Zhang W.N. (2021). Ameliorative effect of SIRT1 in postpartum depression mediated by upregulation of the glucocorticoid receptor. Neurosci. Lett..

[226] Libert S., Pointer K., Bell E., Das A., Cohen D., Asara J., Kapur K., Bergmann S., Preisig M., Otowa T. (2011). SIRT1 Activates MAO-A in the Brain to Mediate Anxiety and Exploratory Drive. Cell.

[227] Li W., Guo B., Tao K., Li F., Liu Z., Yao H., Feng D., Liu X. (2019). Inhibition of SIRT1 in hippocampal CA1 ameliorates PTSD-like behaviors in mice by protections of neuronal plasticity and serotonin homeostasis via NHLH2/MAO-A pathway. Biochem. Biophys. Res. Commun..

[228] Wang Z., Gu J., Wang X., Xie K., Luan Q., Wan N., Zhang Q., Jiang H., Liu D. (2013). Antidepressant-like activity of resveratrol treatment in the forced swim test and tail suspension test in mice: The HPA axis, BDNF expression and phosphorylation of ERK. Pharmacol. Biochem. Behav..

[229] Ji L.L., Ye Y., Nie P.Y., Peng J.B., Fu C.H., Wang Z.Y., Tong L. (2019). Dysregulation of miR-142 results in anxiety-like behaviors following single prolonged stress. Behav. Brain Res..

[230] Li G., Wang G., Shi J., Xie X., Fei N., Chen L., Liu N., Yang M., Pan J., Huang W. (2018). trans-Resveratrol ameliorates anxiety-like behaviors and fear memory deficits in a rat model of post-traumatic stress disorder. Neuropharmacology.

[231] Tseilikman V.E., Shatilov V.A., Zhukov M.S., Buksha I.A., Epitashvily A.E., Lipatov I.A., Aristov M.R., Koshelev A.G., Karpenko M.N., Traktirov D.S. (2023). Limited Cheese Intake Paradigm Replaces Patterns of Behavioral Disorders in Experimental PTSD: Focus on Resveratrol Supplementation. Int. J. Mol. Sci..

[232] Juliani P.Z., Rodrigues T., Bressan G.N., Camponogara C., Oliveira S.M., Brucker N., Fachinetto R. (2024). Effects of association between resveratrol and ketamine on behavioral and biochemical analysis in mice. J. Neural Transm..

[233] Bautista-Aguilera O.M., Alonso J.M., Catto M., Iriepa I., Knez D., Gobec S., Marco-Contelles J. (2022). N-Hydroxy-N-Propargylamide Derivatives of Ferulic Acid: Inhibitors of Cholinesterases and Monoamine Oxidases. Molecules.

[234] Medvedev A. (2004). Tribulin and Endogenous MAO-Inhibitory Regulation In Vivo. Neurotoxicology.

[235] Peng Y., Rideout D.A., Rakita S.S., Gower W.R., You M., Murr M.M. (2010). Does LKB1 Mediate Activation of Hepatic AMP-Protein Kinase (AMPK) and Sirtuin1 (SIRT1) After Roux-en-Y Gastric Bypass in Obese Rats?. J. Gastrointest. Surg..

